# High frequency of melanoma in *cdkn2b^-/-^*/*tp53^-/-^ Xenopus tropicalis*

**DOI:** 10.7150/thno.97475

**Published:** 2024-11-04

**Authors:** Rensen Ran, Lanxin Li, Peng Cheng, Hongyi Li, Huanhuan He, Yonglong Chen, Jing Hang, Weizheng Liang

**Affiliations:** 1 State Key Laboratory of Female Fertility Promotion, Center for Reproductive Medicine, Department of Obstetrics and Gynecology, Peking University Third Hospital, Beijing, China.; 2 Department of Biology, School of Life Sciences, Southern University of Science and Technology, 518055, Shenzhen, China.; 3 Department of Neurosurgery, The second affiliated hospital of Xi'an Medical University, 710119, Xi'an, China.; 4 The School of Medical Technology and Engineering, Fujian Medical University, 350004, Fuzhou, Fujian, China.; 5 Guangdong Provincial Engineering Research Center of Molecular Imaging, The Fifth Affiliated Hospital of Sun Yat-sen University, 519000, Zhuhai, China;; 6 Central Laboratory, The First Affiliated Hospital of Hebei North University, 075000, Zhangjiakou, Hebei, China.

**Keywords:** *CDKN2A*, hereditary melanoma, *cdkn2b* knockout, *tp53* knockout, *Xenopus tropicalis*

## Abstract

**Rationale:** Melanoma, the deadliest form of skin cancer characterized by high therapy resistance, has undergone extensive investigation through the utilization of BRAF^V600E^-driven melanoma animal models. However, there exists a paucity of animal models for the rare hereditary melanoma resulting from germline *CDKN2A* mutations.

**Methods:** Here, employing CRISPR/Cas9 technology, we generated *cdkn2b^-/-^/tp53^-/-^ Xenopus tropicalis* on a *tp53* knockout background to model human *CDKN2A* germline mutation-induced hereditary melanoma.

**Results:** The findings unveiled that *cdkn2b^-/-^/tp53^-/-^* frogs spontaneously developed melanoma, pancreatic cancer, and other tumors. Specifically, these frogs exhibited a high penetrance of spontaneous melanoma, sharing characteristics with melanomas in human hereditary melanoma caused by germline *CDKN2A* mutations. During melanoma development in *cdkn2b^-/-^/tp53^-/-^* frogs, the occurrences of epithelial-to-mesenchymal transition, the reactivation of pigment cell progenitor cell transcriptional states, and the activation in the MAPK, NF-kB, PI3K-Akt, and TGF-β signaling pathways were noted.

**Conclusions:** Overall, *cdkn2b^-/-^/tp53^-/-^ Xenopus tropicalis* provides a vertebrate model for investigating the development of *CDKN2A* germline mutation-induced hereditary melanoma, contributing to the exploration of the pathogenesis of hereditary melanoma in humans.

## Background

Hereditary melanoma, a rare variant of melanoma, arises from the malignant transformation of melanocytes, constituting approximately 10% of all melanomas, exhibiting distinctive traits such as formidable drug resistance, dismal prognostic outcomes, and high mortality rates 1-3. *CDKN2A* germline mutations stand as the predominant genetic alterations associated with hereditary melanoma, accounting for approximately 40% of cases within this subtype 2. However, the pathogenesis of *CDKN2A* germline mutation-induced hereditary melanoma (*CDKN2A*-HM) remains elusive, with a dearth of approved targeted therapies 3-5. Alongside the constraints in clinical research due to ethical limitations on human experimentation, the scarcity of animal models recapitulating the disease's progression poses an additional obstacle 4-6. Therefore, the development of animal models specifically tailored to *CDKN2A*-HM would be highly beneficial for elucidating the intricacies of disease progression and underlying molecular mechanisms.

*CDKN2A* encodes two proteins, p14^ARF^ (p19^ARF^ in mice) and p16^INK4a^ 7. p16^INK4a^ inhibits CDK4/6-directed phosphorylation of retinoblastoma (RB) family proteins, allowing the hypophosphorylated RB binding E2F to govern a G1 cell-cycle arrest 8, 9. p14^ARF^ can execute tumor suppressor activity through inactivating the MDM2 protein to stabilize TP53 8. Currently available data indicate that germline *CDKN2A* loss of function alterations in hereditary melanoma patients mainly affect either p16^INK4a^ alone or both p16^INK4a^ and p14^ARF^, rarely affect p14^ARF^ only 10-12. Thus, the deactivation of the RB and TP53 signaling pathways emerges as a fundamental determinant culminating in the genesis and progression of *CDKN2A*-HM 13. Notably, some instances characterized by the homozygous deletion of large segments within the *CDKN2A* locus may concomitantly entail the excision of the contiguous *CDKN2B* locus, housing the coding sequence for p15^INK4b^ 14-16. Similarly, p15^INK4b^ inhibits CDK4/6-mediated RB phosphorylation, thereby overseeing the cell cycle 14, 17. Nonetheless, the mechanisms underpinning melanoma development in *CDKN2A*-HM remain elusive, as defects in cell cycle regulation, oxidative stress response, and senescence upon *CDKN2A* and *CDKN2B* inactivation could collectively contribute to melanoma susceptibility 10, 18, 19.

At present, there is an absence of validated mammalian models that intravitally recapitulate *CDKN2A*-HM development 6. Within the mammalian genome, as illustrated by mice, the *Cdkn2a* locus encodes two discrete proteins, namely p16^INK4a^ and p19^ARF^, while *Cdkn2b* encodes p15^INK4b^ 20. *Cdkn2a* knockout mice with either selective inactivation of p19^ARF^ (*Arf*^-/-^) or inactivation of both p16^INK4a^ and p19^ARF^ (*Ink4a*^-/-^/*Arf*^-/-^) failed to develop melanomas 21, 22. Intriguingly, mice lacking intact *Ink4a* but retaining one functional *Arf* allele developed melanomas with low penetrance 23. Compound knockout of *Cdkn2a* and *Cdkn2b* (deficient for p15^INK4b^, p16^INK4a^ and p19^ARF^) or *Cdkn2a* and *Tp53* in mice did increase tumor predisposition in general, but did not promote melanoma development 20, 24. In fish and amphibian species, the absence of the *cdkn2a* gene is counterbalanced by the presence of *cdkn2ab* in fish and *cdkn2b* in amphibians 25-27. Both of these counterparts encode the p15^Ink4b^ protein and demonstrate a marked degree of genetic conservation in contrast to mammalian p16^INK4a^ or p15^INK4b^ 27. Consequently, a regulatory framework governed by p15^Ink4b^ mediates the modulation of the Rb signaling pathway in these organisms. Nevertheless, *cdkn2ab^-/-^* medaka fail to undergo spontaneous tumorigenesis, while *tp53^-/-^* zebrafish exhibit an exceedingly scant occurrence of spontaneous melanoma (below 0.2%) 25, 28, 29. No fish with simultaneous knockout of *cdkn2ab* and *tp53* has been reported yet.

*Xenopus tropicalis*, a representative diploid amphibian model, manifests a genomic homology of 79% with the human genome 30. Our previous investigations have revealed an approximate 20% penetrance of spontaneous melanoma in *tp53^-/-^ Xenopus tropicalis*, effectively mirroring the trajectory of human cutaneous melanoma development 31. Although in *Xenopus tropicalis*, it remains to be determined whether a compound homozygous deletion of *rb1*, *rbl1*, and *tp53* can lead to melanoma development, highly penetrant excessive skin pigmentation was observed in *rb1* and *rbl1* double mosaic mutants with various *tp53* targeting backgrounds 32. Collectively, we hypothesized that engineering *cdkn2b^-/-^*/*tp53^-/-^ Xenopus tropicalis* atop the *tp53^-/-^* foundation, thus inducing the disruption of Rb and Tp53 signaling, could model the intricate course of *CDKN2A*-HM development in this animal model. Here, leveraging the *tp53^-/-^ Xenopus tropicalis* as a foundation, we generated *cdkn2b^-/-^*/*tp53^-/-^ Xenopus tropicalis* lines via the CRISPR/Cas9 technique. Remarkably, at 18 months, *cdkn2b^-/-^*/*tp53^-/-^ Xenopus tropicalis* shows a spontaneous melanoma penetrance of 58%, escalating to an 81% penetrance at 30 months. This study further elucidates the transcriptional state of melanocyte progenitor cells during spontaneous melanoma progression in *cdkn2b^-/-^*/*tp53^-/-^ Xenopus tropicalis*. In conclusion, the* cdkn2b^-/-^/tp53^-/-^ Xenopus tropicalis* serves as a vertebrate model that partially models the development of human *CDKN2A*-HM *in vivo*. Additionally, it provides a valuable platform for investigating the complex molecular mechanisms involved in melanoma initiation and progression.

## Methods

### *Xenopus tropicalis* maintenance and husbandry

Adult *Xenopus tropicalis* frogs were purchased from Nasco (Fort Atkinson, WI, USA; http://www.enasco.com). Frog maintenance and husbandry followed the established method, and fertilized embryos of *Xenopus tropicalis* were obtained using the same approach 31. All *Xenopus tropicalis* experiments were approved by the Institutional Animal Care and Use Committee at the Southern University of Science and Technology.

### *In vitro* transcription, embryo microinjection, genotyping and quantitative real-time PCR (qPCR)

The pCS2-Sp9Cas9 plasmid underwent *Not* I linearization and transcription based on the protocol outlined in the mMessage mMachine SP6 Kit (Ambion, Austin, TX, USA). The *in vitro* transcription of guide RNAs (gRNAs) followed the instructions provided by the Transcript Aid T7 High Yield Transcription Kit (Thermo Fisher Scientific, Rockford, IL, USA). The expression constructs for *cdkn2b* gRNA utilized the pUC57-T7-gRNA scaffold vector. The specific target sequence for the *cdkn2b* gRNA is 5'-CCCTGTAAATGCCACAAACTCCC-3'. The gRNA template was generated by subjecting the pUC57-T7-gRNA scaffold to PCR amplification using the Trac-reverse primer and a gRNA forward primer that includes a T7 promoter and the target sequence for the *cdkn2b* gRNA. Please refer to Supplemental Table S1 for the primer sequences. The purification of Cas9 mRNA and gRNAs, as well as the microinjection of *Xenopus tropicalis* embryos, were conducted following the previously described method 33.

To evaluate the efficiency of targeted *cdkn2b* disruption, a pool of five randomly selected healthy embryos was collected 24 h post-microinjection. Genomic DNA was extracted from these embryos, followed by PCR amplification of the targeted region. The amplified PCR products were purified and cloned into the pMD18-T vector (Takara, Katsu, Japan) using TA cloning. Sanger DNA sequencing was performed on randomly chosen single colonies to detect indel mutations. For genotyping, the nails of adult offspring were gently trimmed, and genomic DNA was extracted from the trimmed portion following the instructions provided by the TIANamp Genomic DNA Kit (Tiangen, Beijing, China). Subsequently, PCR amplification was conducted using genotyping primers, and the resulting PCR products were directly subjected to Sanger DNA sequencing. The primer sequences for genotyping PCR were available in Supplemental Table S1. For the qPCR experiments, total RNA was extracted using the TransZol Up Plus RNA Kit (ER501-01, Transgen Biotech). cDNA was synthesized *in vitro* using the TransScript^®^ Uni One-Step gDNA Removal and cDNA Synthesis SuperMix (AU311-03, Transgen Biotech). TransTaq^®^ DNA Polymerase High Fidelity (HiFi) (AP131-11, Transgen Biotech) was used for the qPCR analyses. The specific qPCR primer sequences used in this study are listed in Supplemental Table S1.

### Hematoxylin-Eosin staining, immunofluorescent staining, transmission electron microscopy (TEM)

The samples underwent histological analysis, beginning with fixation in FAS eyeball fixative (Servicebio, Wuhan, China) at room temperature for 24 h. Subsequently, the fixed samples underwent dehydration using graded ethanol (75%, 85%, 95%, and 100%), followed by replacement with xylene and embedding in paraffin wax. Slices with a thickness of 6 μm were prepared from the embedded tissues and subsequently dewaxed in xylene. Rehydration was performed using graded ethanol concentrations (100%, 95%, and 70%) prior to staining with the hematoxylin-eosin staining kit (Baso, Zhuhai, China) and immunofluorescent staining. Hematoxylin-eosin staining and immunofluorescence experiments were carried out exactly according to our previous methods 34. The following primary antibodies were employed: anti-Tyrosinase (ab180753, Abcam), anti-MiTF (ab3201, Abcam). Additionally, two secondary antibodies were utilized: Goat Anti-Mouse IgG H&L (Alexa Fluor^®^ 488) (ab150113, Abcam) and Goat Anti-Rabbit IgG H&L (Alexa Fluor^®^ 594) (ab150080, Abcam). Mounting Medium with DAPI - Aqueous & Fluoroshield (ab104139, Abcam) was employed to stain the cell nucleus. Hematoxylin-eosin-stained results were captured using an Olympus BX53 upright microscope (Olympus, Japan).

The TEM sample preparation method was as follows: *Xenopus tropicalis* tissue samples, sized 1 mm × 1 mm, were fixed overnight at 4 °C in 2% glutaraldehyde. Following fixation, samples underwent four washes of 10 min each with a 10 mM PBS solution. Subsequently, 1% osmium tetroxide was applied to the samples for fixation at room temperature for 3 h. The samples were then subjected to two washes of 10 min each with a 10 mM PBS solution and two washes of 10 min each with ddH_2_O. After rinsing with ddH_2_O, the samples were treated with a 2% uranyl acetate solution at room temperature for 2 h or overnight at 4 °C. Following this, the samples were washed four or more times with ddH_2_O for 10 min each until the rinsed water became clear. Post-rinsing, the samples underwent dehydration with a gradient concentration of acetone: 30% acetone once, 50% acetone once, 75% acetone once, and 100% acetone twice, each for 10 min. Simultaneously with the gradient dehydration, Epoxy resin (9.8 g), DDSA (5.6 g), NMH (4.6 g), and DMP-30 (0.28 mL) were mixed thoroughly at room temperature using a rotary mixer for at least 4 h to prepare a 100% resin mixture. Then, at room temperature, the samples were treated with 25% resin-acetone once, 50% resin-acetone once, 75% resin-acetone once, and 100% resin once, each for 1.5-2 h. Following these steps, new 100% resin was added, and the samples were left overnight at 4 °C. Unused 100% resin was sealed with parafilm and stored at 4 °C for later use. The next day, the samples were embedded in new 100% resin. Subsequently, the embedded samples were baked in a 60 °C oven for 2 days to complete the preparation of TEM samples for ultrathin sectioning. Finally, the prepared TEM samples were sent to Wuhan Servicebio Technology Co., Ltd. for ultrathin sectioning, TEM observation, and photography.

### RNA-seq library preparation and RNA-seq analysis

Samples were collected from 18-month-old tropical clawed frogs, including spontaneously formed abdominal benign nevi and their adjacent tissues, abdominal dysplastic nevi, and invasive melanoma on the back with corresponding adjacent tissues. Each group comprised two replicates. The purpose of collecting these samples was to extract total RNA for the subsequent preparation of RNA-seq libraries. Following the grinding of tissue samples in a mortar with liquid nitrogen, total RNA extraction was carried out according to the manufacturer's instructions for TransZol Up lysis reagent (ET111-01, TransGen Biotech). Subsequently, the extracted total RNA samples were submitted to Wuhan Frasergen Information Co., Ltd. for RNA-seq library preparation and sequencing using the MGI high-throughput sequencer. Wuhan Frasergen Information Co., Ltd. also conducted the RNA-seq analysis. RNA-seq data analysis methods followed the protocol previously published by our group 35, 36. Briefly, SOAPnuke software (v2.1.0) was employed for filtering raw reads to obtain clean reads. The essential parameters were configured as follows: -lowQual=20, -nRate=0.005, and -qualRate=0.5, while all other parameters retained their default values. The filtering process included excluding paired reads with adapter sequences, eliminating paired reads with an N (indicating indeterminate base information) proportion exceeding 0.5%, and removing low-quality paired reads, where bases with a Qphred value not exceeding 20 constituted over 50% of the entire read length. The clean reads underwent alignment to the *Xenopus tropicalis* reference genome UCB_Xtro_10.0 via HISAT2. Subsequently, Bowtie2 software was employed to map the quality-controlled sequences to the reference transcriptome. RSEM analyzed the Bowtie2 alignment results, determining the number of reads mapped to each transcript for every sample and calculating FPKM (Fragments Per Kilobase per Million bases) values. Differential expression analysis employed DESeq2, considering genes with a P-value < 0.05 and an absolute log2(fold change) > 1 as differentially expressed. DESeq2 uses a negative binomial distribution model and Wald test or LRT test to calculate P-values. Gene Ontology (GO) and Kyoto Encyclopedia of Genes and Genomes (KEGG) enrichment analyses utilized the R package clusterProfiler version 4.2.2, with P values guiding the identification of pertinent biological process terms and KEGG pathways. RNA-seq data has been publicly deposited to Genome Sequence Archive (https://ngdc.cncb.ac.cn/gsa/) with the accession number CRA015502.

### Statistics and reproducibility

Data were analyzed using GraphPad Prism version 9 (GraphPad Prism Software, Inc., CA, USA) and expressed as means ± standard deviation (SD). Statistical analyses for multiple comparisons were performed using ordinary one-way ANOVA; "ns" indicates no significant difference, * denotes a P-value < 0.05, ** indicates a P-value < 0.01, *** indicates a P-value < 0.001, and **** indicates a P-value < 0.0001. Except for bulk RNA-seq studies, reproducibility was maintained through a minimum of three biological replicates. The accompanying figure legend provides detailed information on the data processing methodology.

## Results

### Feasibility of modeling human CDKN2A-HM in cdkn2b^-/-^/tp53^-/-^ Xenopus tropicalis

**Figure 1:**
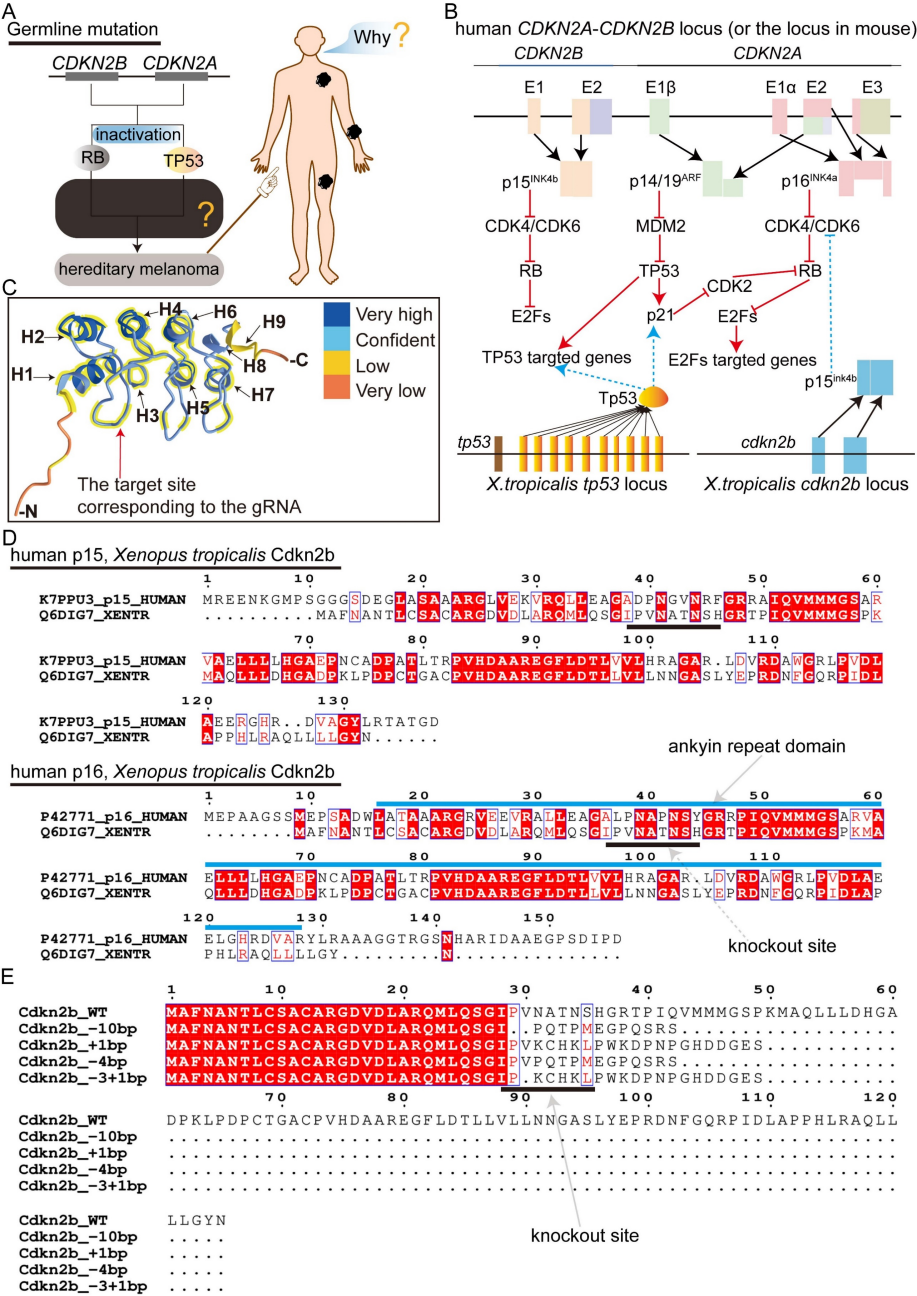
The strategy for modeling human *CDKN2A*-HM in *Xenopus tropicalis*. (A) Inactivating mutations of* CDKN2A* lead to the disruption of the RB and TP53 signaling pathways, which contributes to the onset and progression of *CDKN2A*-HM. However, the molecular mechanisms that regulate the onset and progression of *CDKN2A*-HM are still not fully understood. (B) The proteins encoded by the *Xenopus tropicalis cdkn2b* gene (p15^Ink4b^) and the *tp53* gene (Tp53) govern signaling pathways that are evolutionarily conserved with those regulated by the human *CDKN2A*-*CDKN2B* locus, which encodes the proteins P14^ARF^, P15^INK4b^, and P16^INK4a^. (C) Structural analysis reveals conserved sequences in *Xenopus tropicalis* p15^Ink4b^ and human P16^INK4a^ proteins, with helical structures numbered H1-H9. The conserved domains are highlighted with thick yellow lines. Additionally, spatial information for guide RNA targeting the *cdkn2b* gene is illustrated in this panel. (D) A comparative analysis was performed between the p15^Ink4b^ (Cdkn2b) encoded by *Xenopus tropicalis cdkn2b* and the human *CDKN2B*-encoded p15^INK4b^ (p15), as well as the human *CDKN2A*-encoded p16^INK4a^ (p16). The diagram illustrates the locations of the ankyrin repeat domain and gRNA target sites. The amino acid sequence data for these proteins were sourced from the UniProt database (https://www.uniprot.org/). (E) The prediction of *Xenopus tropicalis* Cdkn2b protein for non-triplet mutant Indels. The figure indicates the gRNA knockout sites.

**Figure 2:**
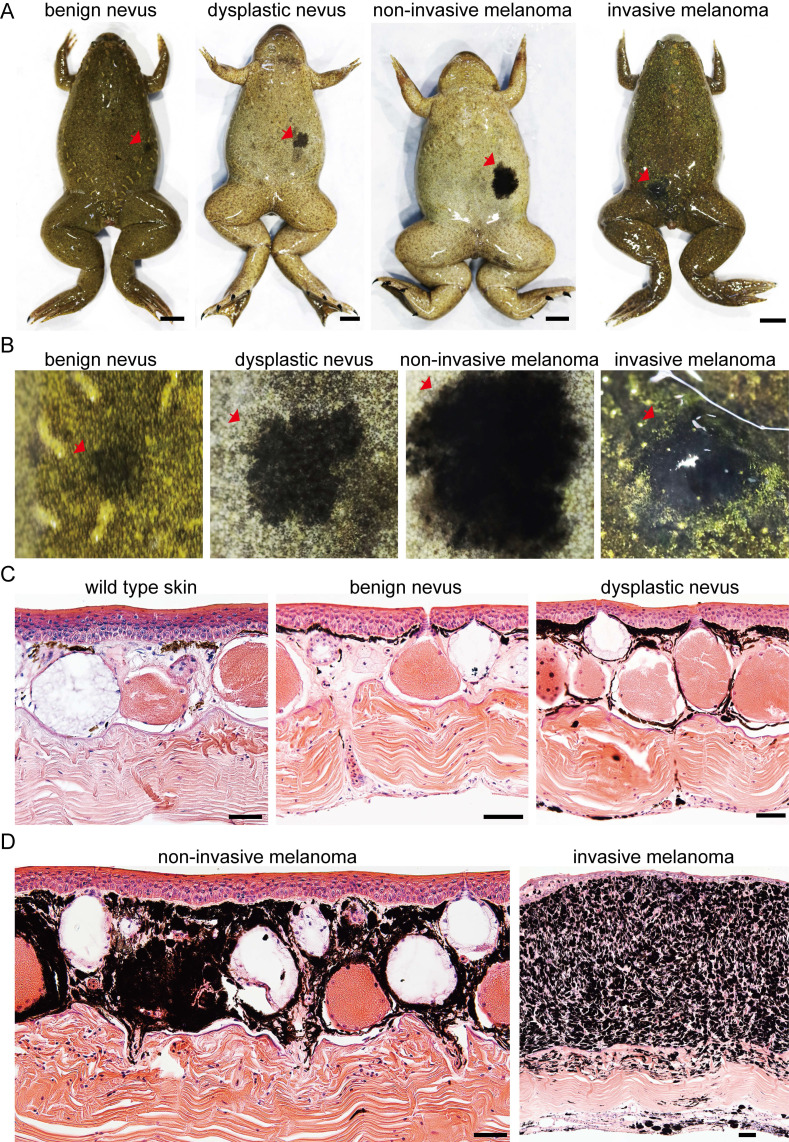
Development of *cdkn2b^-/-^/tp53^-/-^ Xenopus tropicalis* melanoma. (A) Representative images of benign nevus, dysplastic nevus, non-invasive melanoma, and invasive melanoma spontaneously generated in 18-month-old *cdkn2b^-/-^/tp53^-/-^ Xenopus tropicalis*, with a total of 14 frogs observed. For specific lesion details, please refer to Supplementary Table 2. Red arrows indicate the lesion locations. (B) Enlarged images of the corresponding lesions in Panel (A). Red arrows indicate the lesion locations. Please refer to the scale bar in Panel A for the size of Panel (B). (C) and (D), H&E staining results of dorsal skin samples from the same batch of wild type *Xenopus tropicalis* and corresponding lesion samples shown in Panel (B). The images display representative staining results from 10 tissue sections. Scale bars: 5 mm for (A), 50 μm for (C) and (D).

The targeted deletion of *Cdkn2a* in vertebrates presents an optimal melanoma model for recapitulating the progression of *CDKN2A*-HM. Nonetheless, the literature currently lacks descriptions of mice and zebrafish models capable of replicating the progression of *CDKN2A*-HM (Figure S1A). In *Xenopus tropicalis*, the sole identified homologous gene to human *CDKN2A* is *cdkn2b*, with the *cdkn2b* gene in* Xenopus tropicalis* encoding the p15^Ink4b^ protein (Supplementary Figure 1B). Protein homology analysis reveals substantial similarity between *Xenopus tropicalis* p15^Ink4b^ and human p16^INK4a^ and p15^INK4b^, with slightly higher homology to human p16^INK4a^ (Figure S1B and Figure S2). In humans, deletions in *CDKN2A* lead to the inactivation of the RB and P53 signaling pathways, driving the progression of *CDKN2A*-HM (Figure 1A-B) 13. Therefore, we aimed to simultaneously inactivate the Rb and Tp53 signaling pathways in *Xenopus tropicalis* to model the disease progression of *CDKN2A*-HM (Figure 1A-B and Figure S1A). Although *Xenopus tropicalis* lacks the *cdkn2a* gene, its *cdkn2b*-encoded p15^Ink4b^ shares highly conserved ANKYR functional domains with human p16^INK4a^ (Figure 1C-D). Overall, the conserved amino acid sequences between *Xenopus tropicalis* p15^Ink4b^ and human p16^INK4a^ are predominantly located in α-helix structures, with a smaller portion found in β-sheet structures (Figure 2C and Figure S1B). This underscores the functional conservation between *Xenopus tropicalis*' p15^Ink4b^ and human p16^INK4a^, providing a foundation for potentially replicating the occurrence and progression of human *CDKN2A*-HM in *Xenopus tropicalis*. Furthermore, the Tp53 ortholog in *Xenopus tropicalis* shows high conservation with human TP53, as demonstrated in Supplementary Figure 3. In our previous research, we successfully established the *tp53^-/-^ Xenopus tropicalis* line 31. Therefore, we propose that generating a *cdkn2b^-/-^/tp53^-/-^ Xenopus tropicalis* line could serve as a promising model for replicating the molecular mechanisms underlying the development and progression of human *CDKN2A*-HM in this species.

To generate the *cdkn2b^-/-^/tp53^-/-^ Xenopus tropicalis* line upon the existing *tp53^-/-^* foundation, initial efforts involved crossbreeding *tp53^-/-^ Xenopus tropicalis* with wild-type counterparts, yielding fertilized eggs with a *tp53^+/-^* genotype. Following this, guide RNA targeting *cdkn2b* and Cas9 mRNA were microinjected into approximately 200 uncleaved *tp53^+/-^* fertilized eggs to effectuate *cdkn2b* knockout. The contemporary G0 mosaic *cdkn2b* knockout exhibited an efficiency of 93%, resulting in diverse indels, including those with the potential to induce frameshift mutations (Figure S4A). Six months later, 50 sexually mature G0 mosaic *cdkn2b* knockout *Xenopus tropicalis* were obtained. Three randomly selected pairs of G0 adults were bred to produce F1 offspring. As anticipated, the resulting F1 individuals exhibited diverse genotypes for *cdkn2b* and *tp53* (Figure S4B). In CRISPR/Cas9-mediated gene knockout studies, frameshift-inducing indels often result in premature stop codons in the mRNA of the targeted gene. This leads to the production of truncated proteins or activates the nonsense-mediated mRNA decay pathway, causing target mRNA degradation and ultimately resulting in loss of protein function, achieving the goal of gene knockout 37, 38. Our designed *cdkn2b* guide RNA targets the upstream ankyrin repeat domain of the Cdkn2b protein, where induced frameshift mutations lead to the loss of the conventional Cdkn2b protein core structure (Figure 1C-E). Consequently, we label *Xenopus tropicalis* with frameshift-inducing indels mutations in both alleles of *cdkn2b* as *cdkn2b^-/-^* (Figure 1E and Figure S4B). The designation *cdkn2b^+/-^* indicates one allele of *cdkn2b* has been knocked out due to frameshift-inducing indels, while the other allele remains wild type.

Thus, building on the *tp53^-/-^ Xenopus tropicalis* line, we have developed the *cdkn2b^-/-^* and other *Xenopus tropicalis* lines. The *cdkn2b^-/-^/tp53^-/-^ Xenopus tropicalis* line is anticipated to be a valuable model for studying the development and progression of human *CDKN2A*-HM. Subsequently, from the same cohort of F1 individuals, we randomly selected frogs representing nine different genotypes: *cdkn2b^-/-^/tp53^-/-^*, *cdkn2b^+/-^/tp53^-/-^*, *cdkn2b^+/+^/tp53^-/-^*, *cdkn2b^-/-^/tp53^+/-^*, *cdkn2b^+/-^/tp53^+/-^*, *cdkn2b^+/+^/tp53^+/-^*, *cdkn2b^-/-^/tp53^+/+^*, *cdkn2b^+/-^/tp53^+/+^*, and wild type (WT). These frogs are undergoing further husbandry for subsequent experiments.

### High penetrance of spontaneous melanoma formation in *cdkn2b^-/-^/tp53^-/-^ Xenopus tropicalis*

**Figure 3:**
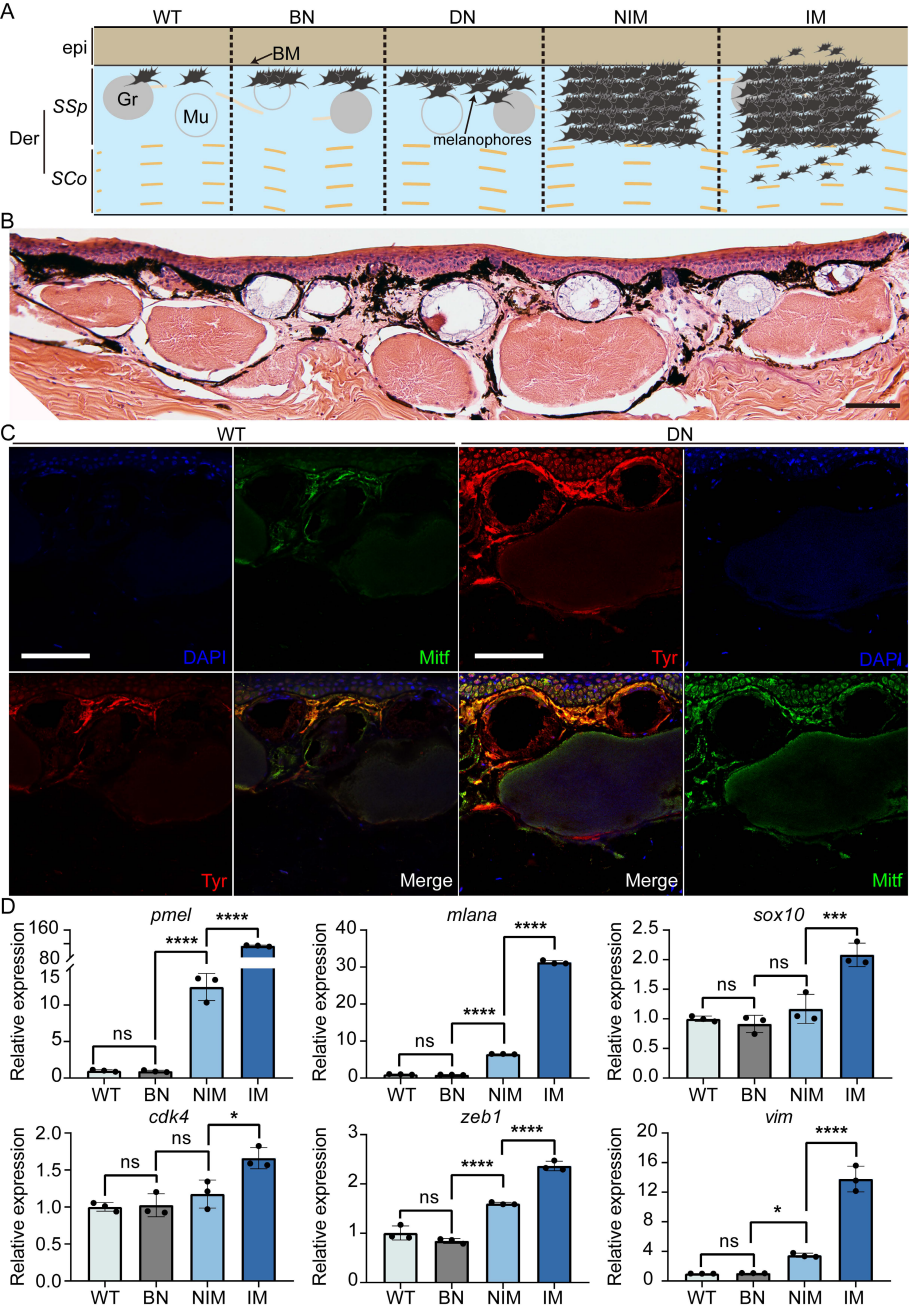
Pathological and molecular characteristics at various melanocytic neoplasms of *cdkn2b^-/-^/tp53^-/-^ Xenopus tropicalis*. (A) Schematic representation illustrating the progression of melanoma in *Xenopus tropicalis*. The designations BN, DN, NIM, and IM correspond to benign nevi, dysplastic nevi, non-invasive melanoma, and invasive melanoma, respectively. Der and epi refer to the dermis and epidermis, while SSp and SCo denote the stratum spinosum and stratum corneum, respectively. BM, Gr, and Mu indicate the basement membrane, granular gland, and mucous gland. For clarity, the schematic presents only select tissue structures within the skin of *Xenopus tropicalis*. (B) Hematoxylin and eosin staining results for a 22-month-old *cdkn2b^-/-^/tp53^-/-^ Xenopus tropicalis* exhibiting dysplastic nevi. (C) The DN (dysplastic nevi) samples show immunofluorescence staining results adjacent to the paraffin sections presented in B, while WT denotes the immunofluorescence results for the dorsal skin of a 22-month-old wild-type *Xenopus tropicalis*. The primary antibodies utilized for immunofluorescence were Tyr and Mitf. "Merge" indicates the merged immunofluorescence results. Scale bars in B and C represent 50 μm, with the images displaying representative staining results from ten tissue sections. (D) qPCR results for marker genes associated with the malignant progression of melanoma (*pmel*, *mlana*, *sox10*, *cdk4*, *zeb1*, and *vim*) in the dorsal skin of a 22-month-old wild-type *Xenopus tropicalis* and age-matched *cdkn2b^-/-^/tp53^-/-^ Xenopus tropicalis* with benign nevi, non-invasive melanoma, and invasive melanoma (n=3). In the statistical analysis, multiple comparisons were conducted using ordinary one-way ANOVA; "ns" indicates no significant difference, * denotes a P-value < 0.05, ** indicates a P-value < 0.01, *** indicates a P-value < 0.001, and **** indicates a P-value < 0.0001.

During frog husbandry, spontaneous nevi and melanoma was observed in various genotypes of *Xenopus tropicalis* at 18 months of age, including *cdkn2b^-/-^/tp53^-/-^*, *cdkn2b^+/-^/tp53^-/-^*, *cdkn2b^+/+^/tp53^-/-^*, *cdkn2b^-/-^/tp53^+/-^*, *cdkn2b^+/-^/tp53^+/-^*, *cdkn2b^+/+^/tp53^+/-^*, and *cdkn2b^-/-^/tp53^+/+^* individuals. The penetrance of nevi and melanoma varied among these genotypes, with *cdkn2b^-/-^/tp53^-/-^* frogs exhibiting the highest penetrance at 58%, significantly surpassing the *tp53^-/-^* cohort (Figure 2, Table 1, and Figure S5). Furthermore, melanoma lesions in *cdkn2b^-/-^/tp53^-/-^* frogs were notably larger and irregularly shaped (Figure 2 and Figure S5), consistent with features observed in human *CDKN2A*-HM 39. The occurrence of melanoma in these frogs showed no gender differences or dorsal-ventral preferences, similar to *tp53^-/-^* frogs' melanoma 31. Histopathological examination revealed that the development process of melanoma in *cdkn2b^-/-^/tp53^-/-^* frogs paralleled that in *tp53^-/-^* frogs 31, characterized by four stages: benign nevi, dysplastic nevi, non-invasive melanoma, and invasive melanoma (Figure 2B-D and Figure 3A), mirroring the progression of human cutaneous melanoma 40. Additionally, we attempted to characterize benign nevi, dysplastic nevi, non-invasive melanoma, and invasive melanoma in *cdkn2b^-/-^/tp53^-/-^ Xenopus tropicalis* using a variety of commercial antibodies that target human melanoma markers (Zeb1, Gpnmb, Vim, Sox10, S100beta, Tyr, Mitf). The results indicated that, except for the Tyr and Mitf antibodies, all other antibodies were ineffective. While the immunofluorescence signals from Tyr and Mitf could identify melanocytes in various melanocytic neoplasms, they could not differentiate between benign nevi, dysplastic nevi, non-invasive melanoma, and invasive melanoma (Figure 3B-C). Ultimately, we characterized the *cdkn2b^-/-^/tp53^-/-^ Xenopus tropicalis* melanocytic neoplasms using qPCR. The results showed that as the melanoma progressed, the expression of malignant progression marker genes (*pmel*, *mlana*, *sox10*, *cdk4*, *zeb1*, and *vim*) significantly increased, with notable expression differences observed among benign nevi, dysplastic nevi, and invasive melanoma (Figure 3D). This supports the applicability of our previous classification criteria to *cdkn2b^-/-^/tp53^-/-^ Xenopus tropicalis* melanoma. Furthermore, the high expression of *sox10*, *cdk4*, *zeb1*, and *vim* suggests that the invasive melanoma in *cdkn2b^-/-^/tp53^-/-^ Xenopus tropicalis* may have undergone dedifferentiation and completed the epithelial-to-mesenchymal transition (EMT).

At 18 months of age, aside from *cdkn2b^-/-^/tp53^-/-^* frogs with spontaneous invasive melanoma at 57%, the majority of other genotypes primarily exhibited spontaneous benign nevi and dysplastic nevi, with only a minority displaying non-invasive melanoma, and no spontaneous occurrence of invasive melanoma (Table S2). The incidence of spontaneous melanoma in this batch of 18-month-old *tp53^-/-^ Xenopus tropicalis* was 25% (Table 1), higher than the previously reported incidence in 14-month-old *tp53^-/-^ Xenopus tropicalis* 31. This clearly illustrates an increasing incidence of melanoma in *tp53^-/-^ Xenopus tropicalis* with prolonged age. The incidence of spontaneous melanoma in *cdkn2b^-/-^/tp53^-/-^* and other genotypes of *Xenopus tropicalis* also increases with prolonged time (Table 1).

Dysplastic nevi from 14-month-old *cdkn2b^-/-^/tp53^-/-^ Xenopus tropicalis* were grafted onto the dorsal region of colorless and immunodeficient *Xenopus tropicalis* (*mitf^-/-^/prkdc^-/-^/il2rg^-/-^*) 34. After a 135-day transplantation period, observation revealed that dysplastic nevi on the colorless and immunodeficient *Xenopus tropicalis* showed no evidence of melanoma cell metastasis and maintained robust survival. Conversely, those transplanted onto colorless *Xenopus tropicalis* (*mitf^-/-^*) underwent complete regression due to immune rejection (Figure S6). This finding underscores the resemblance of *cdkn2b^-/-^/tp53^-/-^ Xenopus tropicalis* dysplastic nevi to their human counterparts, highlighting their limited metastatic potential.

**Figure 4:**
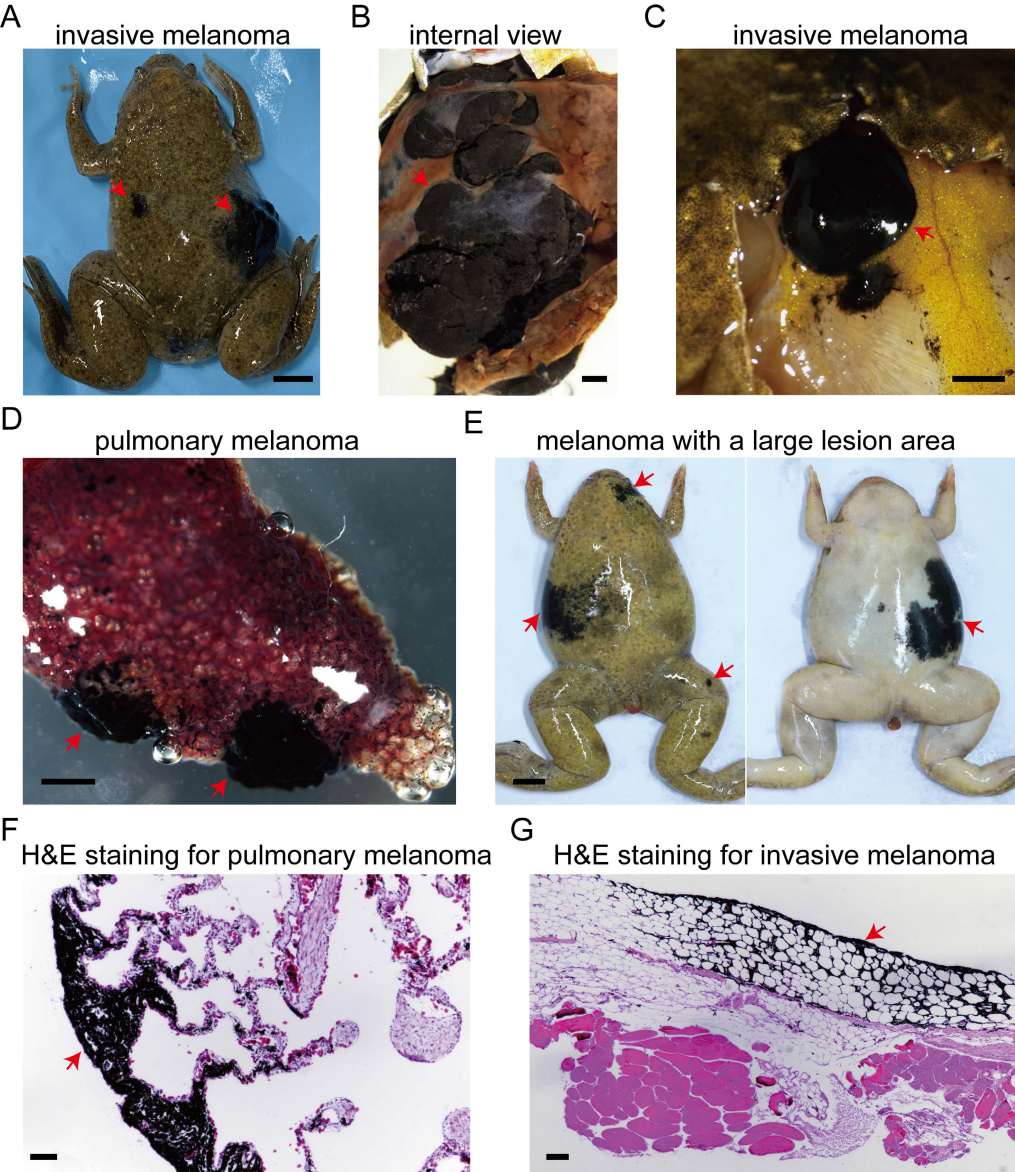
*cdkn2b^-/-^/tp53^-/-^ Xenopus tropicalis* melanoma demonstrates distant metastasis. (A) A 30-month-old *cdkn2b^-/-^/tp53^-/-^ Xenopus tropicalis* spontaneously developed invasive melanoma, which has exhibited distal metastasis. (B) An internal representation of this invasive melanoma, indicated by the red arrow on the right side of Panel (A), reveals melanoma cells proliferating extensively, forming darkly pigmented tissue surrounded by non-pigmented growths. (C) The presence of distant metastasis of the invasive melanoma is highlighted by the red arrow on the left side of Panel (A), with the cancer spreading to the corresponding subcutaneous dorsal fat tissue. (D) Two distinct sites of pulmonary melanoma lesions have been identified in the lungs of *Xenopus tropicalis*, as depicted in Panel A. (E) The characteristic pathological features of 30-month-old *cdkn2b^-/-^/tp53^-/-^ Xenopus tropicalis* melanoma include large lesion areas, irregular shapes, and multiple lesion sites. Four out of 13 spontaneously generated melanomas in *cdkn2b^-/-^/tp53^-/-^ Xenopus tropicalis* exhibit these traits. (F) and (G), Representative hematoxylin and eosin staining results from paraffin-embedded tissue sections of pulmonary melanoma samples (Panel D) and melanoma samples metastasized to subcutaneous dorsal fat tissue (Panel C) are shown, respectively. The images display representative staining results from 10 tissue sections. The red arrow indicates the location of the melanoma lesion. Scale bars: 5 mm for (A) and (E), 1 mm for (B-D), 50 μm for (F) and (G).

In another batch comprising these nine genotypes of *Xenopus tropicalis*, the incidence of spontaneous melanoma reached 81% in 30-month-old *cdkn2b^-/-^/tp53^-/-^* frogs, and 38% in *tp53^-/-^ Xenopus tropicalis* (Table 1). In these *cdkn2b^-/-^/tp53^-/-^ Xenopus tropicalis*, we observed a melanoma exhibiting distant metastasis to the dorsal fat tissue (Figure 4A-C and 4G). Additionally, invasive melanoma was detected in this frog's lung, as depicted in histological sections showing melanoma cells invading the lung tissue (Figure 4D and 4F). At 30 months of age, spontaneous melanocytic neoplasms in *cdkn2b* and *tp53* knockout *Xenopus tropicalis* mainly appear as dysplastic nevi and non-invasive melanoma. Melanomas in *cdkn2b^-/-^/tp53^-/-^* frogs show larger lesion areas, more lesion sites, and irregular growth patterns (Figure 4E). These pathological features resemble some symptoms of familial atypical multiple mole melanoma syndrome (FAMM) resulting from mutations in the human *CDKN2A* gene 41, 42. Interestingly, *cdkn2b^-/-^ Xenopus tropicalis* also exhibit a propensity for spontaneous melanomas, with the incidence rising from 6% at 18 months to 10% at 30 months (Table 1 and Figure S5). This contrasts with the absence of spontaneous melanomas observed in* Cdkn2b^-/-^* mice and *cdkn2ab^-/-^* medaka 6, 20, 24, 25. There have been no spontaneous melanomas observed in *cdkn2b^+/-^ Xenopus tropicalis*, despite the heterozygous loss of *CDKN2A* found in human *CDKN2A*-HM 43. Hence, the spontaneous onset of melanomas in *cdkn2b^+/-^ Xenopus tropicalis* and the carcinogenic capacity of *cdkn2b^+/-^* remain to be further investigation in forthcoming studies. Although *cdkn2b^-/-^/tp53^+/+^ Xenopus tropicalis* can spontaneously develop melanoma, it may not effectively model the progression of *CDKN2A*-HM. First, *cdkn2b^-/-^/tp53^+/+^ Xenopus tropicalis* regulates the malignant transformation of melanocytes theoretically through inactivation of the Rb signaling pathway, whereas *CDKN2A*-HM involves both the RB and TP53 pathways. Second, the low incidence of spontaneous melanoma in *cdkn2b^-/-^/tp53^+/+^ Xenopus tropicalis* complicates the acquisition of melanoma samples for subsequent studies, hindering research efforts. Therefore, our data not only indicate a higher incidence of spontaneous melanoma in* cdkn2b^-/-^/tp53^-/-^ Xenopus tropicalis*, but also suggest that this model can partially replicate the progression of human *CDKN2A*-HM, making it a valuable resource for melanoma research.

In addition to spontaneous melanomas, a variety of tumors arise spontaneously in *cdkn2b* and *tp53* knockout *Xenopus tropicalis*. These encompass pancreatic cancer, lung cancer, liver cancer, sarcoma, splenic tumors, gastric cancer, and ovarian cancer, with spontaneous pancreatic cancer and sarcoma exhibiting a notably higher penetrance rate (Figure S7 and Table S3).

For *cdkn2b^-/-^/tp53^-/-^ Xenopus tropicalis*, we hypothesize that the incidence of tumors in this genotype will be significantly higher than that in other genotypes of *Xenopus tropicalis*. This hypothesis requires further examination involving a larger sample size of frogs to provide supporting evidence (Table S3). The pathological features of spontaneous pancreatic cancer in these *Xenopus tropicalis* correspond to those of human pancreatic ductal adenocarcinoma 44. Severe desmoplastic reactions result in the embedding of tumor ducts within fibrous tissue. The orderly distribution of ducts and acinar structures within lobules observed in the wild-type pancreas is entirely disrupted in pancreatic cancer of *Xenopus tropicalis* (Figure S7I). Human germline mutations in *CDKN2B* predispose individuals to renal cell carcinoma 45. However, renal cell carcinoma lesions have not been detected in *Xenopus tropicalis* with *cdkn2b* knockout. The significant incidence of spontaneous melanomas, pancreatic cancer, and sarcoma observed in *cdkn2b* and *tp53* knockout *Xenopus tropicalis* corresponds with certain manifestations of the FAMM syndrome 15, 31, 42, 46.

Hence, *cdkn2b* and *tp53* knockout *Xenopus tropicalis* provide an animal model to partially recapitulate human FAMM Syndrome. Specifically, *cdkn2b^-/-^/tp53^-/-^ Xenopus tropicalis* represents a vertebrate model for the high penetrance melanoma associated with human *CDKN2A*-HM. This *cdkn2b^-/-^/tp53^-/-^ Xenopus tropicalis* melanoma model holds potential for elucidating the processes and molecular mechanisms involved in the progression of human *CDKN2A*-HM.

### Melanoma dedifferentiation amidst the progression of *cdkn2b^-/-^*/*tp53^-/-^ Xenopus tropicalis* melanoma

**Figure 5:**
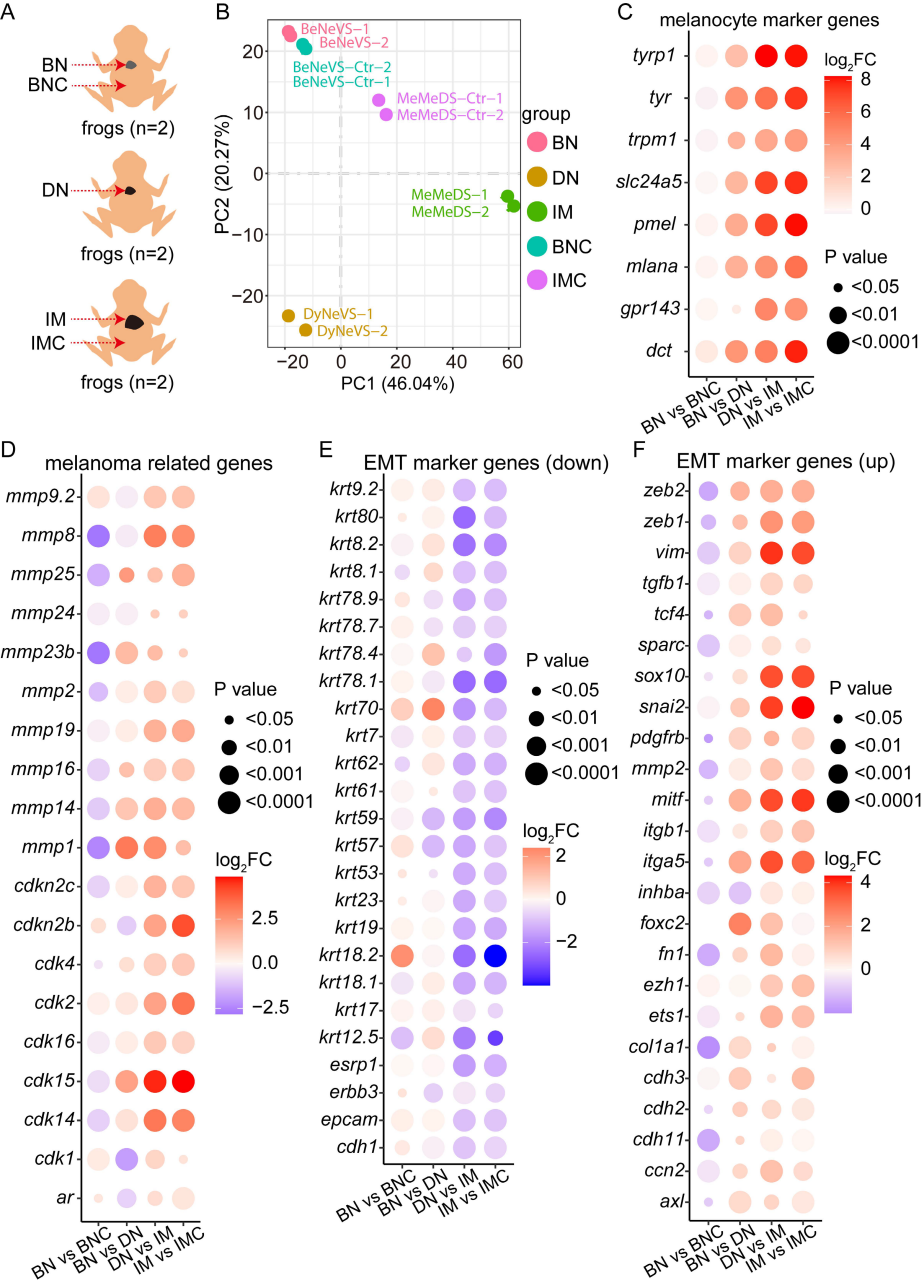
*cdkn2b^-/-^/tp53^-/-^ Xenopus tropicalis* melanoma undergoes epithelial-to-mesenchymal transition (EMT) during its progression. (A) Bulk RNA-seq sampling details during the evolution of melanoma in 18-month-old *cdkn2b^-/-^/tp53^-/-^ Xenopus tropicalis*. Due to the limited availability of *cdkn2b^-/-^/tp53^-/-^ Xenopus tropicalis* melanoma samples, the bulk RNA-seq analysis was performed on benign nevi, dysplastic nevi, and invasive melanoma samples, with two replicates per sample type, each derived from lesions on a single frog. BNC, benign nevi adjacent tissues; BN, benign nevi; DN, dysplastic nevi; IM, invasive melanoma; IMC, adjacent invasive melanoma tissues. "n" represents the number of frogs used for sampling. (B) Principal component analysis results of the Bulk RNA-seq data from **A**, where BeNeVS-Ctr is BNC and MeMeDS-Ctr is IMC. BeVS is BN. DyNeVS is DN. MeMeDS is IM. (C-F) Expression profiles of selected marker genes throughout the melanoma progression. BN vs BNC, BN vs DN, DN vs IM, IM vs IMC indicate comparative analyses between benign nevi adjacent tissues and benign nevi, benign nevi and dysplastic nevi, dysplastic nevi and invasive melanoma samples, adjacent invasive melanoma tissues and invasive melanoma, respectively.

To elucidate the molecular underpinnings of *cdkn2b^-/-^/tp53^-/-^ Xenopus tropicalis* melanoma development, we systematically collected samples including *cdkn2b^-/-^/tp53^-/-^ Xenopus tropicalis* benign nevi adjacent tissues (BNC), benign nevi (BN), dysplastic nevi (DN), invasive melanoma (IM), and adjacent invasive melanoma tissues (IMC) for Bulk RNA-seq analysis (Figure 5A). The transcriptomes of the dorsal and ventral skin of *Xenopus tropicalis* exhibit significant differences, primarily due to the distribution patterns of pigment cells and glands. Consequently, during sampling and data analysis, we compared ventral-derived benign nevi (BN) with peri-nevi (BNC), as well as dorsal-derived invasive melanoma (IM) with adjacent tissues (IMC). Additionally, since primary abnormal proliferation occurs in melanocytes within both nevus and melanoma samples (Figure 2 and Figure 3), we compared ventral-derived dysplastic nevi (DN) with dorsal-derived invasive melanoma (IM). Principal component analysis revealed notable transcriptional disparities among benign nevi, dysplastic nevi, and invasive melanoma; benign nevi exhibited a transcriptional profile akin to benign nevi adjacent tissues, while a more pronounced divergence was observed in the transcriptional profiles of invasive melanoma and its adjacent tissues (Figure 5B). These findings indicate the reproducibility of our sequencing approach and highlight substantial transcriptional alterations occurring during the progression of melanoma.

Further analysis indicated a significant increase in the expression levels of melanocyte marker genes and melanoma-associated genes during the progression of *cdkn2b^-/-^/tp53^-/-^ Xenopus tropicalis* melanoma (Figure 5C-D and Figure S8A-B). This heightened expression of specific matrix metalloproteinases (MMPs) correlates with the phenotype exhibiting a gradually augmented metastatic potential in *cdkn2b^-/-^/tp53^-/-^ Xenopus tropicalis* melanoma (Figure 5D and Figure S8B). The heightened expression of MMPs underscores the imperative to scrutinize the expression profiles of epithelial-to-mesenchymal transition (EMT) marker genes throughout the progression of *cdkn2b^-/-^/tp53^-/-^ Xenopus tropicalis* melanoma 47-49. Consistent with expectations, the expression levels of epithelial state marker genes, including *cdh1* and *epcam*, exhibited marked diminution alongside the progression of *cdkn2b^-/-^/tp53^-/-^ Xenopus tropicalis* melanoma (Figure 5E and Figure S8C). Conversely, the expression levels of mesenchymal state marker genes, such as *cdh2* and *vim*, demonstrated significant elevation during this developmental trajectory (Figure 5F and Figure S8D). Moreover, the upregulation of core EMT transcription factors, notably *zeb2*, *zeb1*, and *snail2*, alongside transcription factors associated with EMT, including *ets1*, *sox10*, and *foxc2*, was conspicuous (Figure 5F and Figure S8D). The noted expression patterns of these aforementioned EMT marker genes collectively signify the occurrence of EMT during the development of *cdkn2b^-/-^/tp53^-/-^ Xenopus tropicalis* melanoma.

**Figure 6:**
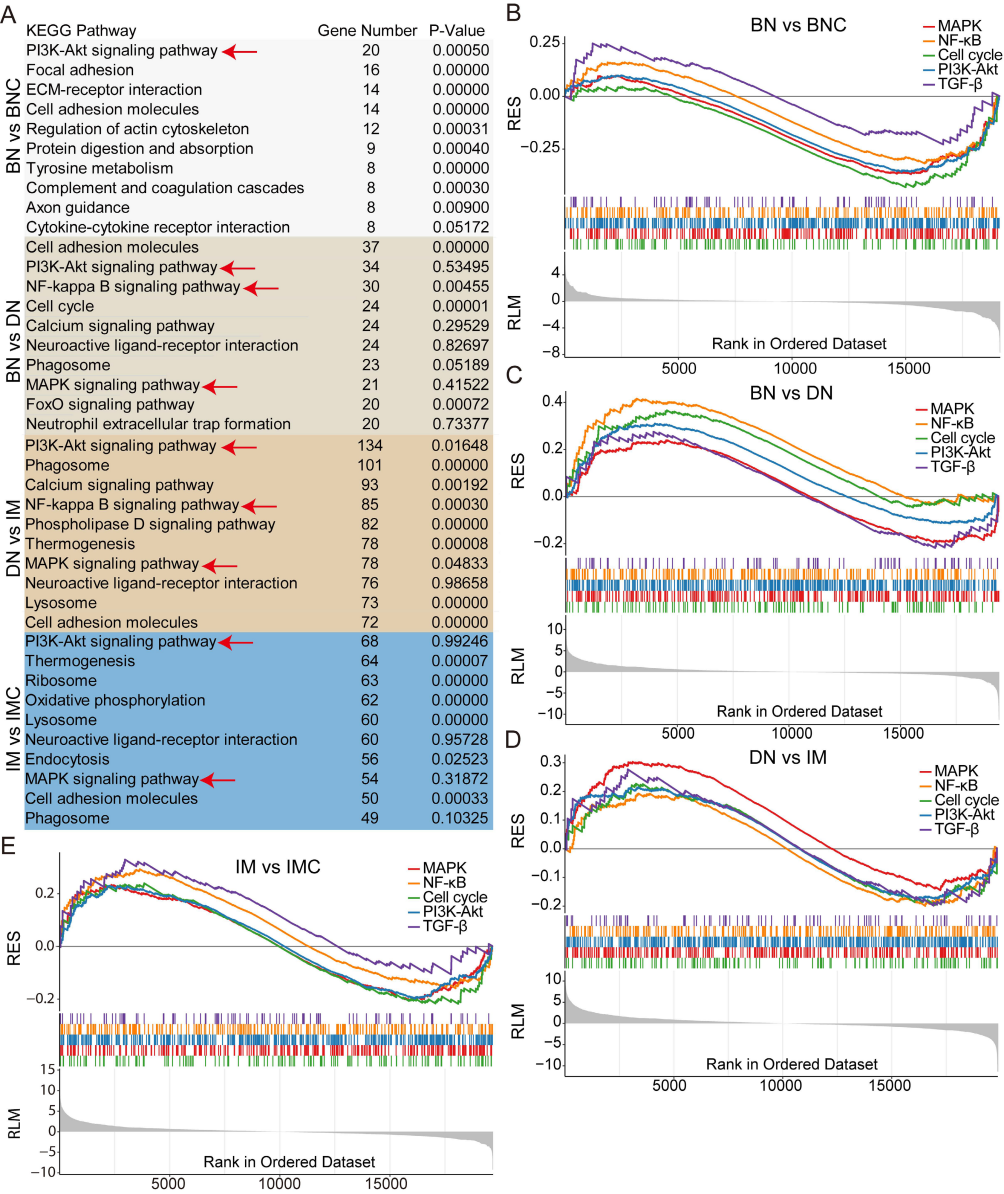
Changes in signaling pathways during the progression of *cdkn2b^-/-^/tp53^-/-^ Xenopus tropicalis*. (A) KEGG analysis results of Bulk RNA-seq during the progression of *cdkn2b^-/-^/tp53^-/-^ Xenopus tropicalis*, with the red arrow pointing to the focused key signaling pathways. (B-E) In the development of benign nevi in *cdkn2b^-/-^/tp53^-/-^ Xenopus tropicalis*, the MAPK, NF-kB, Cell cycle, PI3K-Akt, and TGF-β signaling pathways are downregulated. However, during the progression of benign nevi to malignant melanoma in *cdkn2b^-/-^/tp53^-/-^ Xenopus tropicalis*, the MAPK, NF-kB, Cell cycle, PI3K-Akt, and TGF-β signaling pathways are upregulated. BN vs BNC, BN vs DN, DN vs IM, IM vs IMC indicate comparative analyses between benign nevi adjacent tissues and benign nevi, benign nevi and dysplastic nevi, dysplastic nevi and invasive melanoma samples, adjacent invasive melanoma tissues and invasive melanoma, respectively.

Next, to unveil the activation patterns of signaling pathways during the progression of *cdkn2b^-/-^/tp53^-/-^ Xenopus tropicalis* melanoma, we performed KEGG enrichment analysis on the RNA-seq dataset. This analysis revealed a significant enrichment of the MAPK, NF-kB, PI3K-Akt, and TGF-β signaling pathways throughout the development of *cdkn2b^-/-^/tp53^-/-^ Xenopus tropicalis* melanoma (Figure 6A and Table S4). These pathways displayed a downregulation in benign nevi and an upregulation in dysplastic nevi and invasive melanoma (Figure 6B-E). Notably, the cell cycle signaling pathway displayed a similar pattern, with downregulation in benign nevi and upregulation in dysplastic nevi and invasive melanoma (Figure 6B-E). This pattern aligns with the characteristic melanocyte cell cycle arrest observed in benign nevi, and malignant proliferation of melanocytes seen in dysplastic nevi and invasive melanoma 40. In human *CDKN2A*-mutant melanomas, the disrupted binding of inactive p16^INK4a^ to p65 leads to NF-kB transcriptional activation 50. Concurrently, the inactivation of p14^INK4b^ impedes TP53 function, resulting in heightened IKKα expression and subsequent NF-kB transcriptional activity 50. These suggest a parallel activation of the NF-kB signaling pathway in *cdkn2b^-/-^/tp53^-/-^ Xenopus tropicalis* melanoma, mirroring the molecular features observed in *CDKN2A*-mutant melanomas in humans. Furthermore, the MAPK, PI3K-Akt, and TGF-β signaling pathways are also implicated in human melanoma progression, exerting pivotal regulatory roles 3, 51. These findings illustrate the activation of the MAPK, NF-kB, PI3K-Akt, and TGF-β signaling pathways in *cdkn2b^-/-^/tp53^-/-^ Xenopus tropicalis* melanoma development. Regarding the underlying causes of melanoma development in *cdkn2b^-/-^/tp53^-/-^ Xenopus tropicalis*, although our preliminary whole-genome sequencing study ruled out mutations in *braf*, *nras*, *kras*, *nf1*, *kit*, and *pten* genes, there must be other key driver mutations awaiting discovery. However, elucidating the precise triggers for their activation, their interplay, and their specific regulatory functions in the pathogenesis of *cdkn2b^-/-^/tp53^-/-^ Xenopus tropicalis* melanoma necessitates further investigation in forthcoming studies.

The dedifferentiation of melanoma cells is crucial in the initiation, progression, resistance development, and recurrence of BRAF^V600E^-driven melanoma 3. Thus, does the dedifferentiation of melanoma cells also take place during the progression of melanoma in *cdkn2b^-/-^/tp53^-/-^ Xenopus tropicalis*? To address this inquiry, we scrutinized the expression of marker genes associated with pigment cells in *cdkn2b^-/-^/tp53^-/-^ Xenopus tropicalis* melanomas. Amphibians present a broader range of pigment cell types compared to mammals, including melanophores, xanthophores, and iridophores 52, 53. Notably, the developmental trajectory of pigment cell maturation in *Xenopus tropicalis* exhibits evolutionary conservation akin to zebrafish (Figure S9) 3, 52-54.

**Figure 7:**
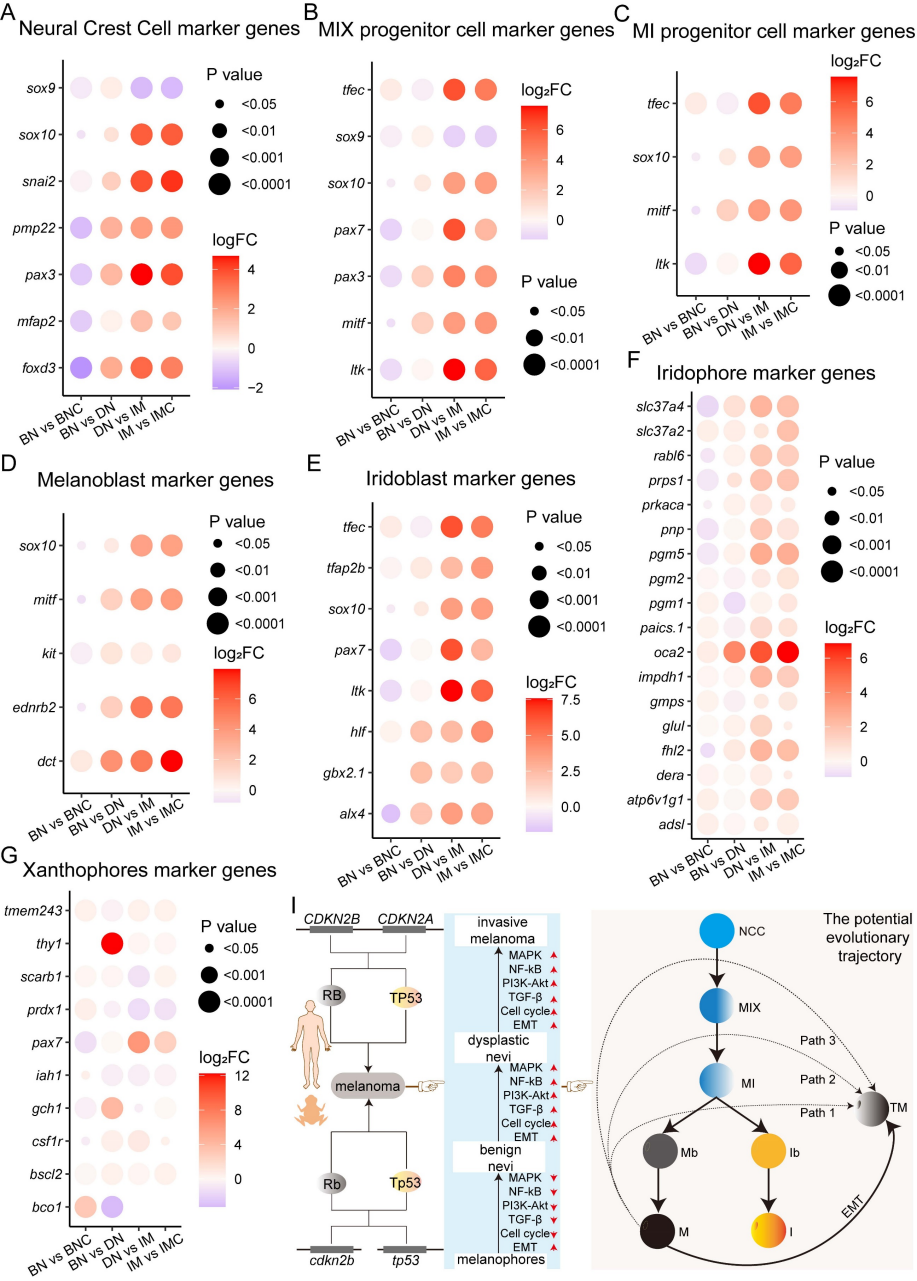
The expression of pigment cell precursor marker genes increases progressively during the development of *cdkn2b^-/-^/tp53^-/-^ Xenopus tropicalis* melanoma. (A-G) Figures present the expression profiles of neural crest cell (NCC), MIX progenitor cell (progenitor cells capable of differentiating into melanophores, xanthophores, and iridophores), MI progenitor cell (progenitor cells capable of differentiating into melanophores and iridophores), melanoblast (Mb), iridoblast (Ib), iridophore (I), and xanthophore (X) marker genes during the progression of *cdkn2b^-/-^/tp53^-/-^ Xenopus tropicalis* melanoma. (I) The schematic diagram provides a graphical summary depicting the establishment of a *cdkn2b^-/-^/tp53^-/-^ Xenopus tropicalis* melanoma model to recapitulate the progression of human *CDKN2A*-HM. The illustration also outlines potential developmental pathways of melanophores and iridophores in *Xenopus tropicalis*, with Path1, Path2, and Path3 indicating potential dedifferentiation routes of *cdkn2b^-/-^/tp53^-/-^ Xenopus tropicalis* melanoma. TM indicates melanoma cells. BN vs BNC, BN vs DN, DN vs IM, IM vs IMC indicate comparative analyses between benign nevi adjacent tissues and benign nevi, benign nevi and dysplastic nevi, dysplastic nevi and invasive melanoma samples, adjacent invasive melanoma tissues and invasive melanoma, respectively.

In *Xenopus tropicalis*, neural crest cells (NCCs) develop into MIX progenitor cells, which are pigment progenitors capable of differentiating into melanophores, iridophores, and xanthophores. These MIX progenitors express specific cellular markers (Figure S9) 54, 55. Further differentiation of MIX progenitors results in three distinct progenitor types: MI (which give rise to melanophores and iridophores), MX (which give rise to melanophores and xanthophores), and IX (which give rise to iridophores and xanthophores). Each of these progenitor types expresses new marker genes while retaining some of the original markers from the MIX progenitors. Consequently, NCCs and MIX, MI, MX, and IX progenitor cells share certain cellular markers. Upon differentiation into mature pigment cells—melanophores, iridophores, and xanthophores—each expresses unique, cell type-specific markers. Consequently, we investigated the expression profiles of marker genes related to NCCs, MIX progenitor cells, MI progenitor cells, and other pigment cells in *cdkn2b^-/-^/tp53^-/-^ Xenopus tropicalis* melanomas (Figure S9). The results demonstrate a significant upregulation in the expression levels of the examined marker genes, excluding *sox9*, as melanoma develops (Figure 7A-C). Particularly noteworthy is the marked increase in expression levels observed for melanoblast marker genes (*sox10*, *mitf*, *kit*, *ednrb2*, and *dct*) and iridoblast marker genes (*tfec*, *tfap2b*, *sox10*, *pax7*, *ltk*, *hlf*, *gbx2.1*, and *alx4*) during melanoma progression (Figure 7D-E). There is a substantial rise in the expression levels of iridophores and melanocytes marker genes throughout the development of *cdkn2b^-/-^/tp53^-/-^ Xenopus tropicalis* melanoma, whereas the upregulation of xanthophores marker genes shows variability during melanoma progression (Figure 7F-G and Figure 5C). These data prompted us to utilize TEM to examine pigment cell alterations, focusing specifically on melanophores, in *cdkn2b^-/-^/tp53^-/-^ Xenopus tropicalis* melanoma samples. TEM analysis revealed a significant proliferation of melanoma cells and a substantial increase in melanosomes during *cdkn2b^-/-^/tp53^-/-^ Xenopus tropicalis* melanoma development (Figure S10). Additionally, by calculating the percentage of the area occupied by proliferating melanocytes, a significant increase in melanocyte proliferation can be observed (Figure S11). Given the conserved developmental pattern of pigment cells in *Xenopus tropicalis* and zebrafish (Figure 7I and Figure S9), it is conceivable that some *Xenopus tropicalis* melanophores and iridophores may derive from MI progenitor cells, which are themselves derived from MIX progenitor cells originating from neural crest cells 3, 52, 53. Hence, in conjunction with the dedifferentiation of melanoma cells into a progenitor cell state as evidenced by the RNA-seq, these results propose that during the progression of *cdkn2b^-/-^/tp53^-/-^ Xenopus tropicalis* melanoma, melanoma cells may transit into pigment cell precursor cells (neural crest cells, MIX progenitor cells, MI progenitor cells, melanoblasts, and iridoblasts), undergo EMT, and subsequently progress into malignant tumor cells exhibiting heightened expression of melanophores and iridophores marker genes.

In conclusion, the progression of *cdkn2b^-/-^/tp53^-/-^ Xenopus tropicalis* melanoma is characterized by the presence of EMT, reactivation of pigment cell precursor cell transcriptional states, and activation of the MAPK, NF-kB, PI3K-Akt, and TGF-β signaling pathways (Figure 7I). Unfortunately, the precise cellular trajectories and molecular mechanisms driving the initiation and progression of *cdkn2b^-/-^/tp53^-/-^ Xenopus tropicalis* melanoma necessitate further investigation in future research endeavors.

## Discussion

Here, we found that *Xenopus tropicalis* with knockout of *cdkn2b* and *tp53* spontaneously develop melanoma, pancreatic cancer, and other tumors, thereby establishing a valuable vertebrate model that partially recapitulates features of human FAMM syndrome. Notably, *cdkn2b^-/-^/tp53^-/-^ Xenopus tropicalis* exhibit a significantly increased incidence of spontaneous melanoma, with lesions resembling those seen in human *CDKN2A*-HM associated with FAMM syndrome. Consequently, this model may serve as a unique platform for investigating the initiation and progression of human *CDKN2A*-HM. However, further research is needed to determine the extent to which *cdkn2b^-/-^/tp53^-/-^ Xenopus tropicalis* melanoma mimics human *CDKN2A*-HM. Additionally, during melanoma progression in these frogs, we observed epithelial-to-mesenchymal transition (EMT), accompanied by the reactivation of pigment cell precursor transcriptional states and activation of key signaling pathways, including MAPK, NF-κB, PI3K-Akt, and TGF-β. Overall, the *cdkn2b^-/-^/tp53^-/-^ Xenopus tropicalis* melanoma model offers a unique platform to delve into the cellular and molecular mechanisms underpinning melanoma dedifferentiation in the context of disrupted Rb and Tp53 signaling pathways. This model holds promise for advancing our understanding of the pathogenesis and therapeutic strategies for human *CDKN2A*-HM.

*CDKN2A* germline mutations associated with FAMM syndrome have been examined in mouse, zebrafish, and medaka animal models, yet a model directly recapitulating FAMM syndrome remains absent 6, 20-25, 28. *Xenopus tropicalis* lacking *cdkn2b* and *tp53* display a heightened incidence of spontaneous melanoma and pancreatic cancer, echoing the pathological traits observed in human FAMM syndrome. Despite the absence of the *cdkn2a*-*cdkn2b* locus in *Xenopus tropicalis*, the presence of the *cdkn2b* locus suggests that concurrent knockout of *cdkn2b* and *tp53* in *Xenopus tropicalis* is akin to triple knockout of *Cdkn2a*, *Cdkn2b*, and *Trp53* in mice, potentially explaining species-specific phenotypic variations 26, 27. In fish, while the *cdkn2a*-*cdkn2b* locus is absent, the *cdkn2ab* locus is present. Nevertheless, knockout of medaka's *cdkn2ab* does not induce spontaneous tumor formation 25. Conversely, the incidence of spontaneous melanoma in 30-month-old *cdkn2b^-/-^ Xenopus tropicalis* stands at 10% (Table 1), suggesting that species-specific factors may contribute to observed phenotypic variations among related mouse, fish, and frog models. In *Xenopus tropicalis*, previous studies have noted that CRISPR/Cas9-mediated knockout of *rb1* and *rbl1* in founder generation crispants with varying *tp53* mutation backgrounds exhibits excessive skin pigmentation 32. However, documentation on the occurrence of spontaneous melanoma in these crispants and their offspring is lacking. Our previous research reveals a notably higher incidence of spontaneous melanoma in *tp53* knockout *Xenopus tropicalis* compared to *tp53* knockout zebrafish, while *Trp53* knockout mice do not develop spontaneous melanoma 31. Hence, these findings imply that the disruption of Rb and Tp53 signaling pathways in *Xenopus tropicalis* predisposes to spontaneous melanoma formation. Unfortunately, the molecular mechanisms underlying this phenomenon warrant further elucidation in future studies.

Following melanoma dedifferentiation, the recurrence of transcriptional states in melanocyte progenitor cells significantly influences melanoma onset, progression, and resistance to treatments 3. It is currently unclear whether melanoma dedifferentiation also occurs during the development of* CDKN2A*-HM 4. In the *cdkn2b^-/-^/tp53^-/-^ Xenopus tropicalis* melanoma model, we observed EMT and heightened expression of pigment cell progenitor cell markers. This suggests that akin to BRAF^V600E^-driven melanoma, during the development of *cdkn2b^-/-^/tp53^-/-^ Xenopus tropicalis* melanoma, melanoma cells undergo dedifferentiation, adopting the transcriptional state of pigment cell progenitors, undergo EMT, culminating in invasive melanoma (Figure 7I). Nevertheless, further investigation is warranted into the melanocyte and melanoma cell heterogeneity, the trajectory of melanocyte malignant transformation, and the underlying molecular mechanisms during *cdkn2b^-/-^/tp53^-/-^ Xenopus tropicalis* melanoma development. The use of single-cell and spatial high-throughput sequencing technologies in *cdkn2b^-/-^/tp53^-/-^ Xenopus tropicalis* melanoma would be highly beneficial in uncovering the mechanisms underlying the malignant progression of melanoma cells 56, 57. Concurrently, pivotal signaling pathways such as MAPK, NF-kB, PI3K-Akt, TGF-β, and the cell cycle are activated in the development of *cdkn2b^-/-^/tp53^-/-^ Xenopus tropicalis* melanoma. Interestingly, these pathways are generally inhibited during benign nevi formation. The reasons behind this inhibition during benign nevi formation, contrasted with their activation and enhancement during benign nevi progression to invasive melanoma, warrant exploration.

In summary, based on the *tp53^-/-^ Xenopus tropicalis*, we utilized CRISPR/Cas9 technology to construct *cdkn2b* and *tp53* knockout *Xenopus tropicalis*. The results revealed that *cdkn2b* and *tp53* knockout *Xenopus tropicalis* spontaneously develop melanoma, pancreatic cancer, and other tumors, making them a valuable vertebrate model that can partially recapitulate features of human FAMM syndrome. The incidence of spontaneously developing melanoma in 2-year-old *cdkn2b^-/-^/tp53^-/-^ Xenopus tropicalis* reaches as high as 81%, and the pathological features of *cdkn2b^-/-^/tp53^-/-^ Xenopus tropicalis* melanoma are similar to those of human *CDKN2A*-HM. Therefore, the *cdkn2b^-/-^/tp53^-/-^ Xenopus tropicalis* melanoma model provides a platform for *in vivo* investigations into the development of *CDKN2A*-HM. This model lays a groundwork for the exploration of therapeutic strategies aimed at hereditary melanoma.

## Supplementary Material

Supplementary figures and tables 1-3.

Supplementary table 4.

## Figures and Tables

**Table 1:** Penetrance of spontaneous melanoma in *Xenopus tropicalis* under different knockout backgrounds of *cdkn2b* and* tp53*

Genotype	18 months^♠^	30 months^♣^
*cdkn2b^+/+^/tp53^+/+^*	0/24 (0%)	0/37 (0%)
*cdkn2b^+/-^/tp53^+/+^*	0/27 (0%)	0/25 (0%)
*cdkn2b^-/-^/tp53^+/+^*	2/32 (6%)	4/39 (10%)
*cdkn2b^+/+^/tp53^+/-^*	5/46 (11%)	6/27 (22%)
*cdkn2b^+/-^/tp53^+/-^*	7/49 (14%)	8/31 (26%)
*cdkn2b^-/-^/tp53^+/-^*	6/28 (21%)	6/11 (55%)
*cdkn2b^+/+^/tp53^-/-^*	3/12 (25%)	13/34 (38%)
*cdkn2b^+/-^/tp53^-/-^*	5/13 (38%)	19/45 (42%)
*cdkn2b^-/-^/tp53^-/-^*	14/24 (58%)	13/16 (81%)

Note: ^♣^ denotes the penetrance of spontaneous melanoma in the first batch of various genotypes of *Xenopus tropicalis*. At this stage, benign nevi and dysplastic nevi are rare, with the majority being *in situ* melanomas and metastatic melanomas. ^♠^ represents the penetrance of spontaneous melanoma in the second batch of F2 generation with various genotypes of *Xenopus tropicalis*. At this stage, the proportions of benign nevi, dysplastic nevi, non-invasive melanomas, and invasive melanomas are detailed in Supplementary Table 2.

## Abbreviations

CDKN2A/Cdkn2a: cyclin-dependent kinase inhibitor 2A
TP53/Trp53/tp53: tumor protein P53
BRAF^V600E^: a V600E amino acid substitution in the B-Raf protein
CRISPR: clustered regularly interspaced short palindromic repeats
Cas9: CRISPR-associated protein 9
MAPK: mitogen-activated protein kinase
NF-kB: nuclear factor kappa-light-chain-enhancer of activated B cells
PI3K-Akt: phosphatidylinositol 3-kinase (PI3K)/protein kinase B (Akt)
TGF-β: transforming growth factor beta
*CDKN2A*-HM: *CDKN2A* germline mutation-induced hereditary melanoma
CDK4/6: cyclin-dependent kinases 4 and 6
RB/Rb: retinoblastoma
E2F: early 2 factor
MDM2: murine double minute 2
CDKN2B/Cdkn2b/cdkn2b: cyclin-dependent kinase inhibitor 2B
RB1: retinoblastoma 1
RBL1: retinoblastoma-like protein 1
TEM: transmission electron microscopy
H&E: Hematoxylin-Eosin staining
ANKYR: Ankyrin repeats
FAMM: familial atypical multiple mole melanoma syndrome
MMPs: matrix metalloproteinases
EMT: epithelial-to-mesenchymal transition

## References

[1] Aoude L, Wadt K, Pritchard A, Hayward N (2015). Genetics of familial melanoma: 20 years after CDKN2A. Pigment Cell Melanoma Res.

[2] Ipenburg N, El Sharouni M, van Doorn R, van Diest P, van Leerdam M, van der Rhee J (2022). Lack of association between CDKN2A germline mutations and survival in patients with melanoma: A retrospective cohort study. J Am Acad Dermatol.

[3] Centeno P, Pavet V, Marais R (2023). The journey from melanocytes to melanoma. Nat Rev Cancer.

[4] Patton E, Mueller K, Adams D, Anandasabapathy N, Aplin A, Bertolotto C (2021). Melanoma models for the next generation of therapies. Cancer cell.

[5] Ionita I, Malita D, Dehelean C, Olteanu E, Marcovici I, Geamantan A (2023). Experimental models for rare melanoma research-the niche that needs to be addressed. Bioengineering (Basel).

[6] Sherr C (2001). Parsing Ink4a/Arf: "pure" p16-null mice. Cell.

[7] Farooq U, Notani D (2022). Transcriptional regulation of INK4/ARF locus by cis and trans mechanisms. Front Cell Dev Biol.

[8] Kim W, Sharpless N (2006). The regulation of INK4/ARF in cancer and aging. Cell.

[9] Sherr CJ (2012). Ink4-Arf locus in cancer and aging. Wiley Interdiscip Rev Dev Biol.

[10] Ming Z, Lim S, Rizos H (2020). Genetic Alterations in the INK4a/ARF Locus: Effects on Melanoma Development and Progression. Biomolecules.

[11] van Doorn R (2023). Surveillance, CDKN2A and survival of familial melanoma. J Eur Acad Dermatol Venereol.

[12] Pissa M, Lapins J, Sköldmark C, Helgadottir H (2023). Melanoma-specific survival before and after inclusion in a familial melanoma dermatologic surveillance program in CDKN2A mutation carriers and non-carriers. J Eur Acad Dermatol Venereol.

[13] Walker G, Hayward N (2002). Pathways to melanoma development: lessons from the mouse. J Invest Dermatol.

[14] Chin L, Pomerantz J, DePinho R (1998). The INK4a/ARF tumor suppressor: one gene-two products-two pathways. Trends Biochem Sci.

[15] Jensen M, Stoltze U, Hansen T, Bak M, Sehested A, Rechnitzer C (2022). CDKN2A/2B9p21.3 Microdeletion involving in a young patient with multiple primary cancers and review of the literature. Cold Spring Harb Mol Case Stud.

[16] Chan A, Han S, Choy W, Beleford D, Aghi M, Berger M (2017). Familial melanoma-astrocytoma syndrome: synchronous diffuse astrocytoma and pleomorphic xanthoastrocytoma in a patient with germline CDKN2A/B deletion and a significant family history. Clin Neuropathol.

[17] Yuan C, Selby T, Li J, Byeon I, Tsai M (2000). Tumor suppressor INK4: refinement of p16INK4A structure and determination of p15INK4B structure by comparative modeling and NMR data. Protein Sci.

[18] Patel S, Wilkinson C, Sviderskaya E (2020). Loss of Both CDKN2A and CDKN2B Allows for Centrosome Overduplication in Melanoma. J Invest Dermatol.

[19] McNeal A, Liu K, Nakhate V, Natale C, Duperret E, Capell B (2015). CDKN2B loss promotes progression from benign melanocytic nevus to melanoma. Cancer Discov.

[20] Krimpenfort P, Ijpenberg A, Song J, van der Valk M, Nawijn M, Zevenhoven J (2007). p15Ink4b is a critical tumour suppressor in the absence of p16Ink4a. Nature.

[21] Serrano M, Lee H, Chin L, Cordon-Cardo C, Beach D, DePinho R (1996). Role of the INK4a locus in tumor suppression and cell mortality. Cell.

[22] Kamijo T, Zindy F, Roussel M, Quelle D, Downing J, Ashmun R (1997). Tumor suppression at the mouse INK4a locus mediated by the alternative reading frame product p19ARF. Cell.

[23] Krimpenfort P, Quon K, Mooi W, Loonstra A, Berns A (2001). Loss of p16Ink4a confers susceptibility to metastatic melanoma in mice. Nature.

[24] Sharpless N, Alson S, Chan S, Silver D, Castrillon D, DePinho R (2002). p16(INK4a) and p53 deficiency cooperate in tumorigenesis. Cancer Res.

[25] Regneri J, Klotz B, Wilde B, Kottler V, Hausmann M, Kneitz S (2019). Analysis of the putative tumor suppressor gene cdkn2ab in pigment cells and melanoma of Xiphophorus and medaka. Pigment Cell Melanoma Res.

[26] Tanaka T, Ochi H, Takahashi S, Ueno N, Taira M (2017). Genes coding for cyclin-dependent kinase inhibitors are fragile in Xenopus. Dev Biol.

[27] Gil J, Peters G (2006). Regulation of the INK4b-ARF-INK4a tumour suppressor locus: all for one or one for all. Nat Rev Mol Cell Biol.

[28] Berghmans S, Murphey R, Wienholds E, Neuberg D, Kutok J, Fletcher C (2005). tp53 mutant zebrafish develop malignant peripheral nerve sheath tumors. Proc Natl Acad Sci U S A.

[29] Patton E, Widlund H, Kutok J, Kopani K, Amatruda J, Murphey R (2005). BRAF mutations are sufficient to promote nevi formation and cooperate with p53 in the genesis of melanoma. Curr Biol.

[30] Hellsten U, Harland R, Gilchrist M, Hendrix D, Jurka J, Kapitonov V (2010). The genome of the Western clawed frog Xenopus tropicalis. Science.

[31] Ran R, Li L, Shi Z, Liu G, Jiang H, Fang L (2022). Disruption of tp53 leads to cutaneous nevus and melanoma formation in Xenopus tropicalis. Mol Oncol.

[32] Naert T, Dimitrakopoulou D, Tulkens D, Demuynck S, Carron M, Noelanders R (2020). RBL1 (p107) functions as tumor suppressor in glioblastoma and small-cell pancreatic neuroendocrine carcinoma in Xenopus tropicalis. Oncogene.

[33] Guo X, Zhang T, Hu Z, Zhang Y, Shi Z, Wang Q (2014). Efficient RNA/Cas9-mediated genome editing in Xenopus tropicalis. Development.

[34] Ran R, Li L, Xu T, Huang J, He H, Chen Y (2024). Revealing mitf functions and visualizing allografted tumor metastasis in colorless and immunodeficient Xenopus tropicalis. Commun Biol.

[35] Niu L, Shen W, Shi Z, Tan Y, He N, Wan J (2021). Three-dimensional folding dynamics of the Xenopus tropicalis genome. Nat Genet.

[36] Shi Z, Liu G, Jiang H, Shi S, Zhang X, Deng Y (2023). Activation of P53 pathway contributes to Xenopus hybrid inviability. Proc Natl Acad Sci U S A.

[37] Popp MW, Maquat LE (2016). Leveraging Rules of Nonsense-Mediated mRNA Decay for Genome Engineering and Personalized Medicine. Cell.

[38] Brogna S, Wen J (2009). Nonsense-mediated mRNA decay (NMD) mechanisms. Nat Struct Mol Biol.

[39] Bishop JN, Harland M, Randerson-Moor J, Bishop DT (2007). Management of familial melanoma. Lancet Oncol.

[40] Miller A, Mihm M (2006). Melanoma. N Engl J Med.

[41] Rashid S, Gupta S, McCormick S, Tsao H (2022). New insights into melanoma tumor syndromes. JID Innov.

[42] Soura E, Eliades P, Shannon K, Stratigos A, Tsao H (2016). Hereditary melanoma: Update on syndromes and management: Emerging melanoma cancer complexes and genetic counseling. J Am Acad Dermatol.

[43] van der Wilk B, Noordman B, Atmodimedjo P, Dinjens W, Laheij R, Wagner A (2020). Development of esophageal squamous cell cancer in patients with FAMMM syndrome: Two clinical reports. Eur J Med Genet.

[44] Neesse A, Algül H, Tuveson D, Gress T (2015). Stromal biology and therapy in pancreatic cancer: a changing paradigm. Gut.

[45] Jafri M, Wake N, Ascher D, Pires D, Gentle D, Morris M (2015). Germline Mutations in the CDKN2B Tumor Suppressor Gene Predispose to Renal Cell Carcinoma. Cancer Discov.

[46] Kimura H, Klein A, Hruban R, Roberts N (2021). The role of inherited pathogenic CDKN2A variants in susceptibility to pancreatic cancer. Pancreas.

[47] Yang J, Antin P, Berx G, Blanpain C, Brabletz T, Bronner M (2020). Guidelines and definitions for research on epithelial-mesenchymal transition. Nat Rev Mol Cell Biol.

[48] Caramel J, Papadogeorgakis E, Hill L, Browne G, Richard G, Wierinckx A (2013). A switch in the expression of embryonic EMT-inducers drives the development of malignant melanoma. Cancer cell.

[49] Pedri D, Karras P, Landeloos E, Marine J, Rambow F (2022). Epithelial-to-mesenchymal-like transition events in melanoma. FEBS J.

[50] Ueda Y, Richmond A (2006). NF-kappaB activation in melanoma. Pigment Cell Res.

[51] Shain A, Joseph N, Yu R, Benhamida J, Liu S, Prow T (2018). Genomic and transcriptomic analysis reveals incremental disruption of key signaling pathways during melanoma evolution. Cancer cell.

[52] Mort R, Jackson I, Patton E (2015). The melanocyte lineage in development and disease. Development.

[53] Subkhankulova T, Camargo Sosa K, Uroshlev L, Nikaido M, Shriever N, Kasianov A (2023). Zebrafish pigment cells develop directly from persistent highly multipotent progenitors. Nature communications.

[54] Joudi EM, Ali N, Nadine T, Muriel C, Hussein F-K, Pierre T (2024). Xenopus as a model system for studying pigmentation and pigmentary disorders. Pigment Cell Melanoma Res.

[55] Alessandro B, E Elizabeth P (2024). Melanocyte lineage dynamics in development, growth and disease. Development.

[56] Longqi L, Ao C, Yuxiang L, Jan M, Holger H, Xun X (2024). Spatiotemporal omics for biology and medicine. Cell.

[57] Itay T, Mario L S (2024). Cancer cell states: Lessons from ten years of single-cell RNA-sequencing of human tumors. Cancer Cell.

