# Supplementary material for: High frequency of melanoma in cdkn2b-/-/tp53-/- Xenopus tropicalis
Source: Theranostics. 2024 Nov 4;14(19):7470–87. doi: 10.7150/thno.97475 (PMC11626935; doi:10.7150/thno.97475)
Supplement: Supplementary file 1 — Supplementary figures and tables 1-3. [file thnov14p7470s1.pdf]

Supplementary Materials

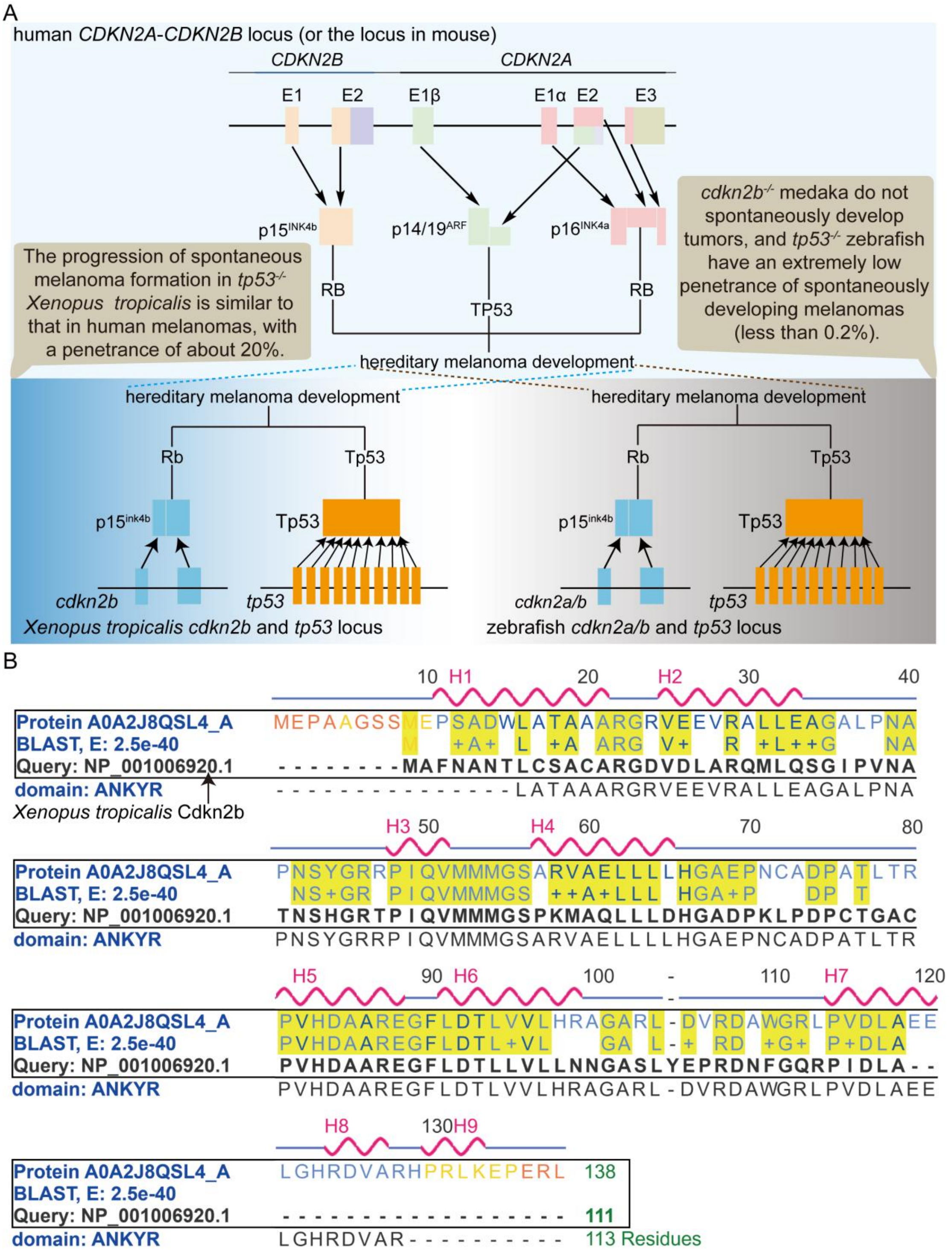

Figure S1. Modeling human *CDKN2A*-HM in *Xenopus tropicalis*. (A) The vertebrate Rb and TP53 signaling pathways in relation to in vivo animal models of melanoma associated with *CDKN2A* germline mutations. *CDKN2A* mutations result in the inactivation of the RB and TP53 signaling pathways.

However, in both mice and zebrafish, there are currently no established animal models that effectively simulate the in vivo progression of melanoma driven by *CDKN2A* germline mutations through Rb or TP53 pathway inactivation. (B) Homology alignment between *Xenopus tropicalis* p15^{Ink4b} (Cdkn2b) and human P16^{INK4a} demonstrates significant similarity, with helical structures numbered H1-H9. The conserved domains are highlighted with thick yellow lines. The data were retrieved from https://www.ncbi.nlm.nih.gov/.

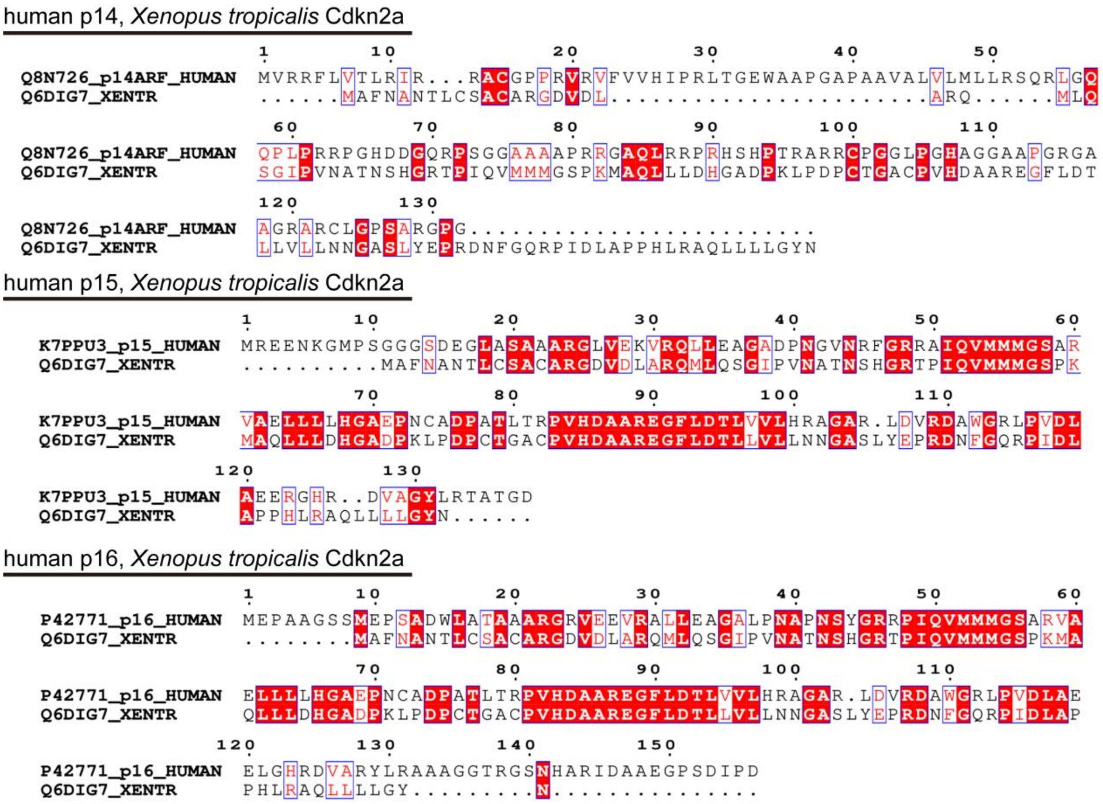

Figure S2. Comparison of the homology between *Xenopus tropicalis* Cdkn2b and human p14, p15, and p16. The amino acid sequence data for these proteins were sourced from the UniProt database (https://www.uniprot.org/).

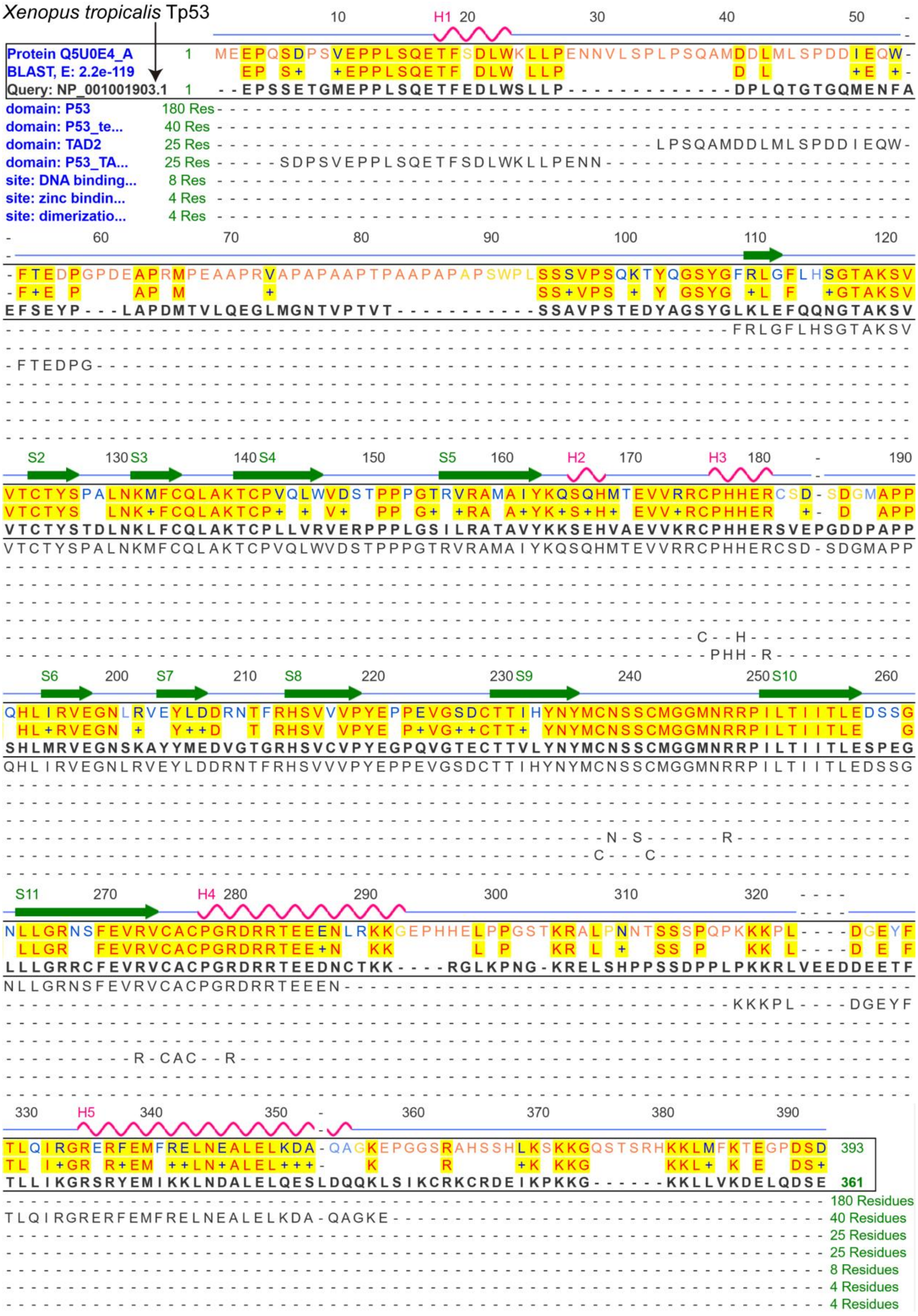

Figure S3. A comparative analysis was conducted to assess the homology between *Xenopus tropicalis* Tp53 and human TP53 proteins. The data was retrieved from https://www.ncbi.nlm.nih.gov/.

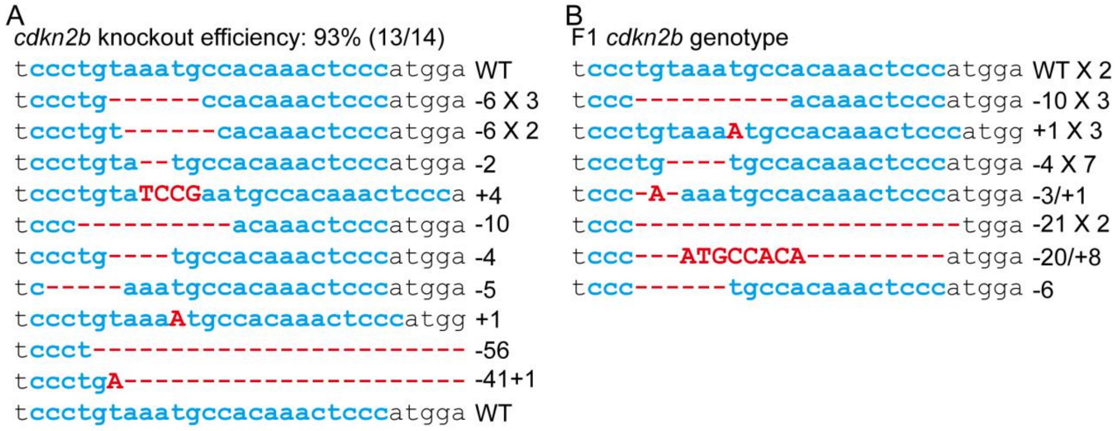

Figure S4. The knockout of *cdkn2b* in *Xenopus tropicalis*. (A) The efficiency of the knockout for contemporary mosaic *Xenopus tropicalis cdkn2b* was determined to be 93%, accompanied by the identification of indels resulting from the knockout process. (B) Genotypes of the F1 generation *Xenopus tropicalis* were examined following the crossing of contemporary mosaic *cdkn2b* knockout frogs. '**N**' denotes the total occurrences of each genotype detected out of 20 tested tadpoles.

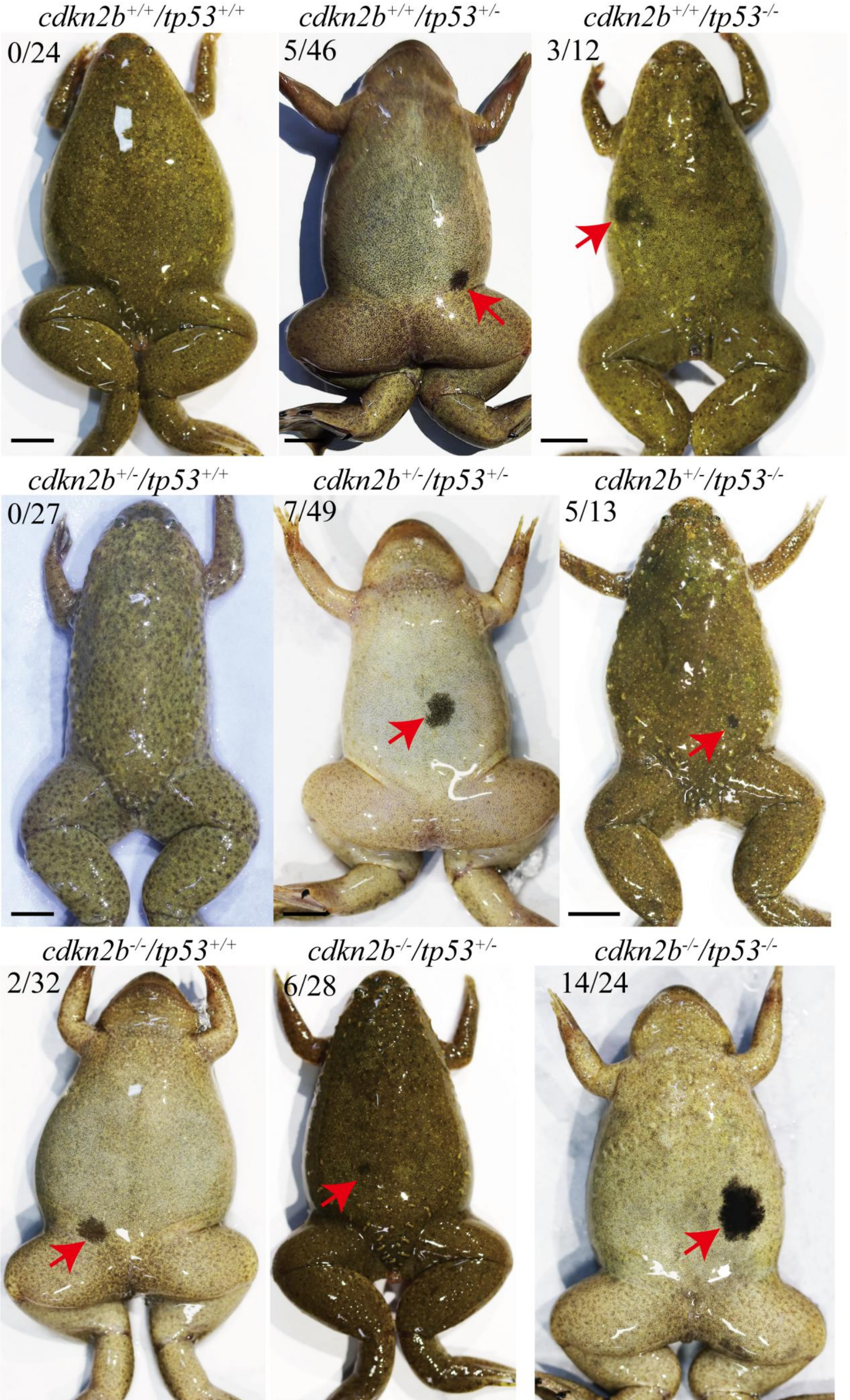

cdkn2b+/+/tp53+/+
0/24
cdkn2b+/+/tp53+/-
5/46
cdkn2b+/+/tp53-/-
3/12
cdkn2b+/-/tp53+/+
0/27
cdkn2b+/-/tp53+/-
7/49
cdkn2b+/-/tp53-/-
5/13
cdkn2b-/-/tp53+/+
2/32
cdkn2b-/-/tp53+/-
6/28
cdkn2b-/-/tp53-/-
14/24

Figure S5. Spontaneous melanoma formation in 18-month-old *Xenopus tropicalis* with *cdkn2b* and *tp53* knockouts. Representative images depict the nevi or melanomas spontaneously developed by each genotype of these frogs. Arrows indicate melanoma lesion locations, with accompanying numbers representing the proportion of frogs developing melanomas out of the total examined. The scale bar is 5 mm.

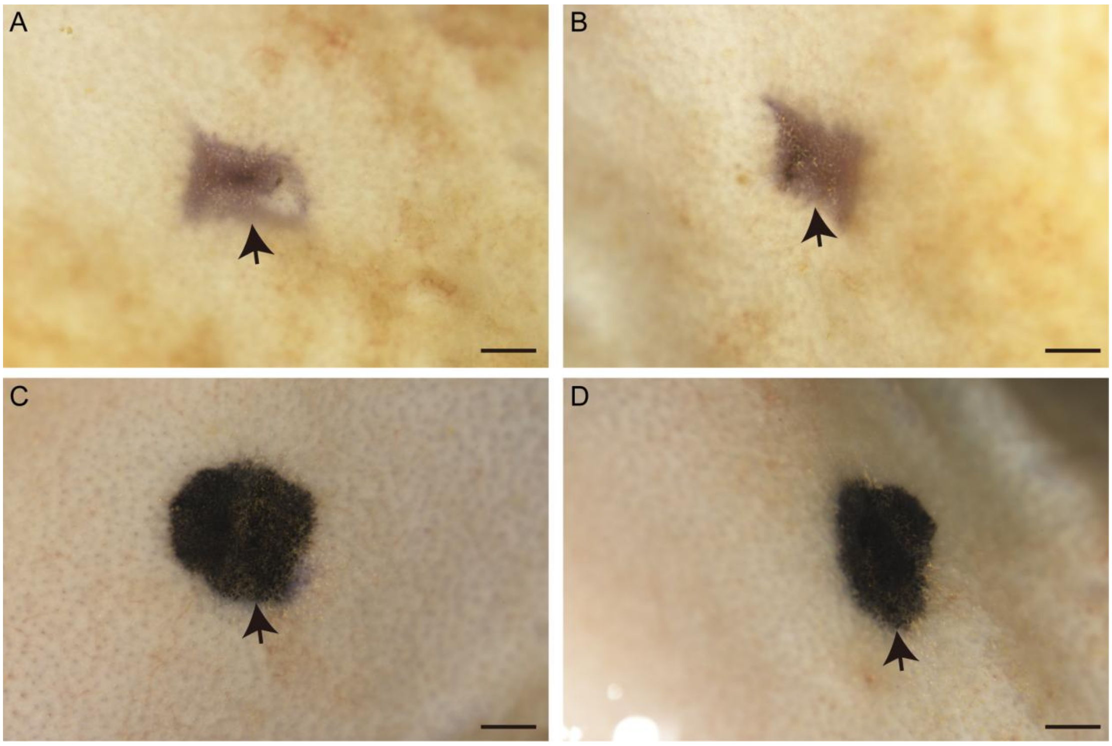

Figure S6. Allograft results of 14-month-old *cdkn2b*$^{-/-}$/*tp53*$^{-/-}$ *Xenopus tropicalis* dysplastic nevi. Images (A-B) depict observations 135 days post-transplantation of dysplastic nevi into recipient *mitf-/- Xenopus tropicalis* (two replicates). Images (C-D) illustrate observations 135 days post-transplantation of dysplastic nevi into recipient *mitf*$^{-/-}$/*prkdc*$^{-/-}$/*il2rg*$^{-/-}$ *Xenopus tropicalis* (two replicates). Black arrows indicate transplanted dysplastic nevi. Scale bars: 1 mm.

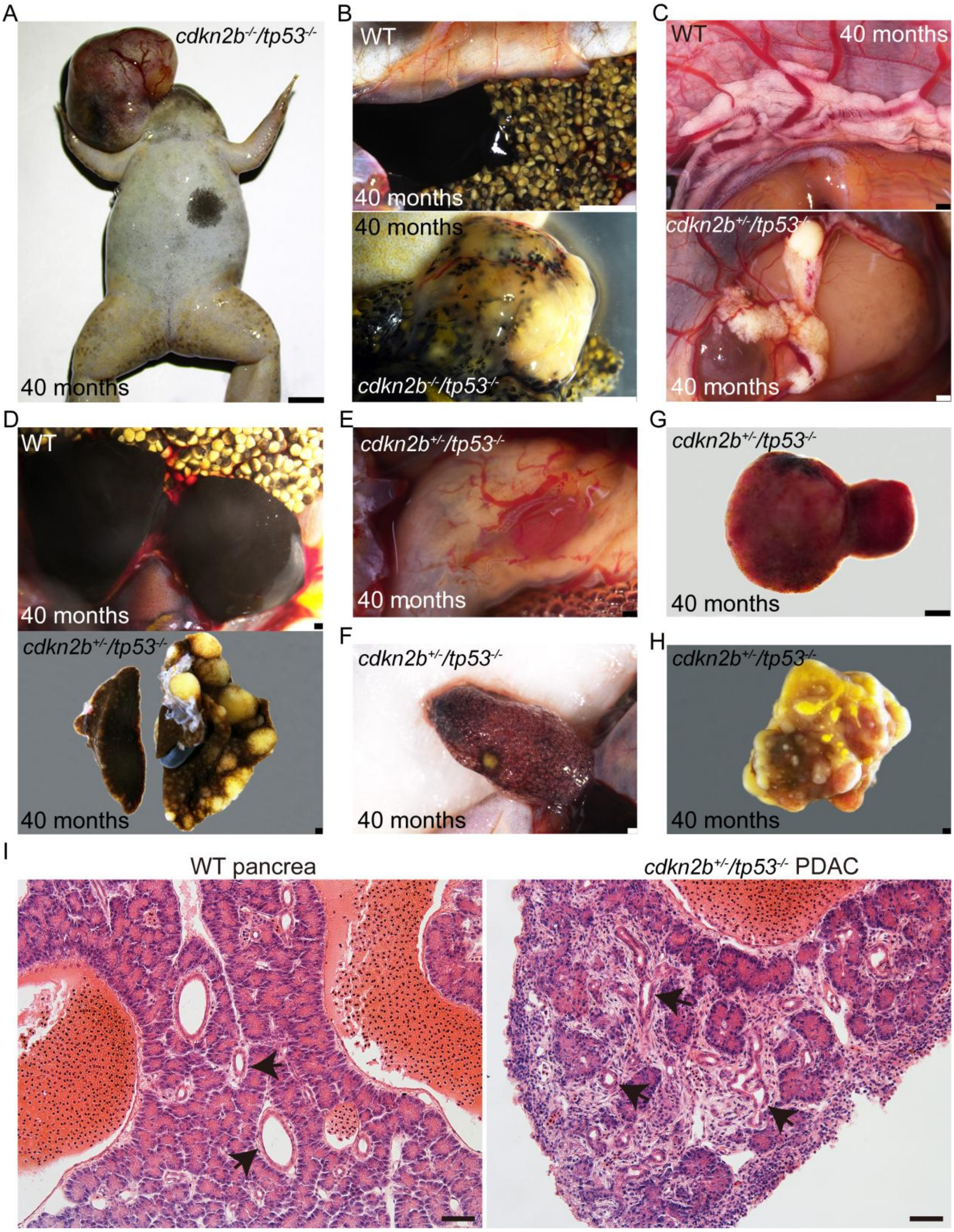

Figure S7. Spontaneous tumors in 40-month-old *Xenopus tropicalis* under different knockout backgrounds of *cdkn2b* and *tp53*, excluding melanoma. Panels (A-H) exhibit representative histopathological images showcasing a spectrum of tumors including sarcoma, ovarian cancer, pancreatic cancer, liver cancer, gastric cancer, lung cancer, splenic tumor, and thoracic sarcoma within the same age cohort of *Xenopus tropicalis*. Panel (I) displays the hematoxylin

and eosin staining results of pancreatic tissue sections comparing WT (wild-type) *Xenopus tropicalis* to *cdkn2b*$^{-/-}$/*tp53*$^{-/-}$ *Xenopus tropicalis* with PDAC (pancreatic ductal adenocarcinoma). The results presented are based on 10 consecutive sections. Scale bars: 5 mm for A, 1 mm for B, 0.5 mm for C-H, and 50 μm for I.

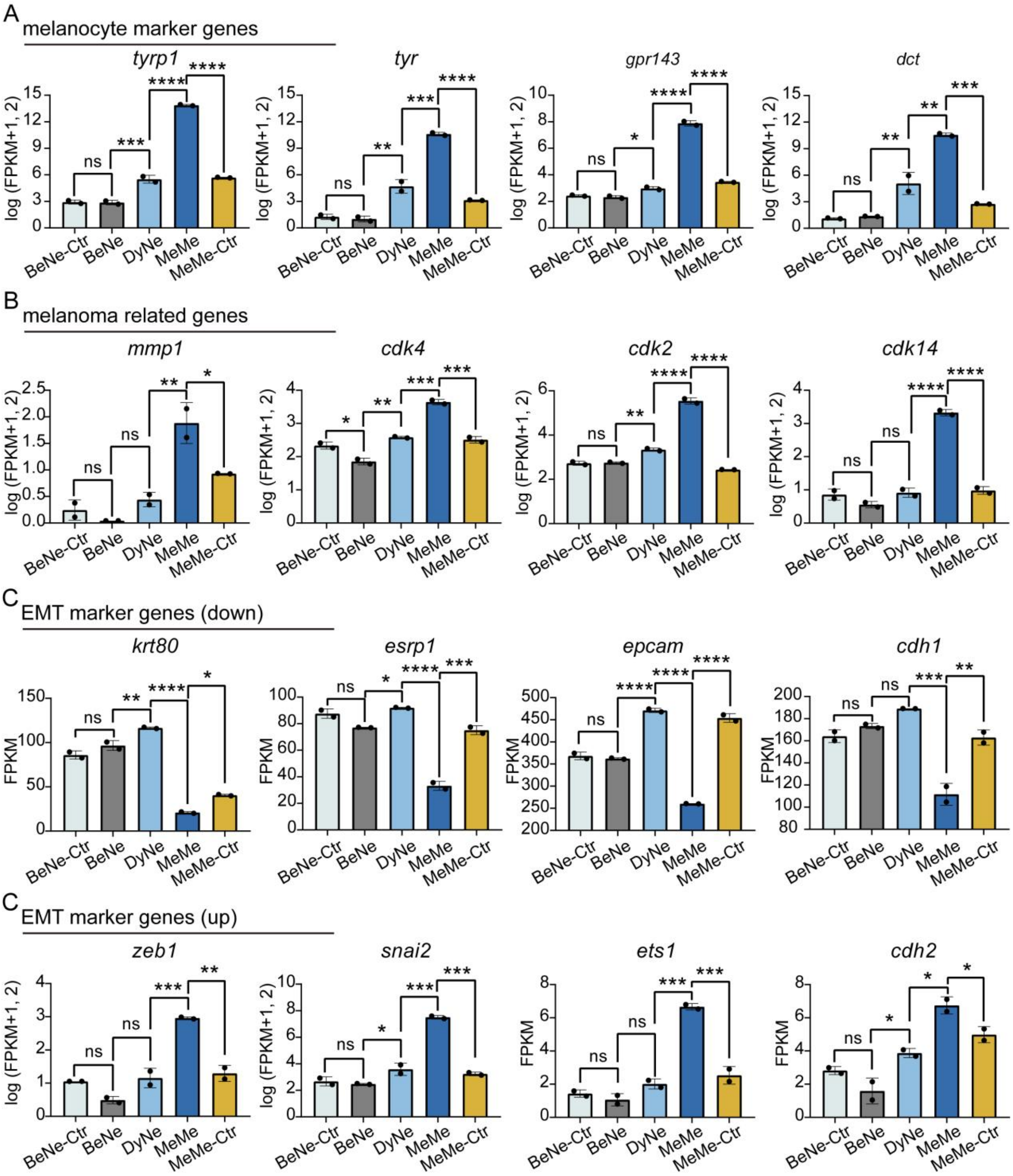

Figure S8. Molecular characteristics of *cdkn2b$^{-/-}$/tp53$^{-/-}$ Xenopus tropicalis* melanoma. The differential expression of melanocyte marker genes (A), melanoma-related genes (B), EMT-downregulated marker genes (C), and EMT-upregulated marker genes (D) in *cdkn2b$^{-/-}$/tp53$^{-/-}$ Xenopus tropicalis* benign nevi, dysplastic nevi, and invasive melanoma samples is shown using FPKM values detected in bulk RNA-seq data. BeNe-Ctr, benign nevi adjacent tissues; BeNe, benign nevi; DyNe, dysplastic nevi; MeMe, invasive melanoma; MeMe-Ctr, adjacent invasive melanoma tissues. In the statistical analysis,

multiple comparisons were conducted using ordinary one-way ANOVA; "ns" indicates no significant difference, * denotes a P-value < 0.05, ** indicates a P-value < 0.01, *** indicates a P-value < 0.001, and **** indicates a P-value < 0.0001.

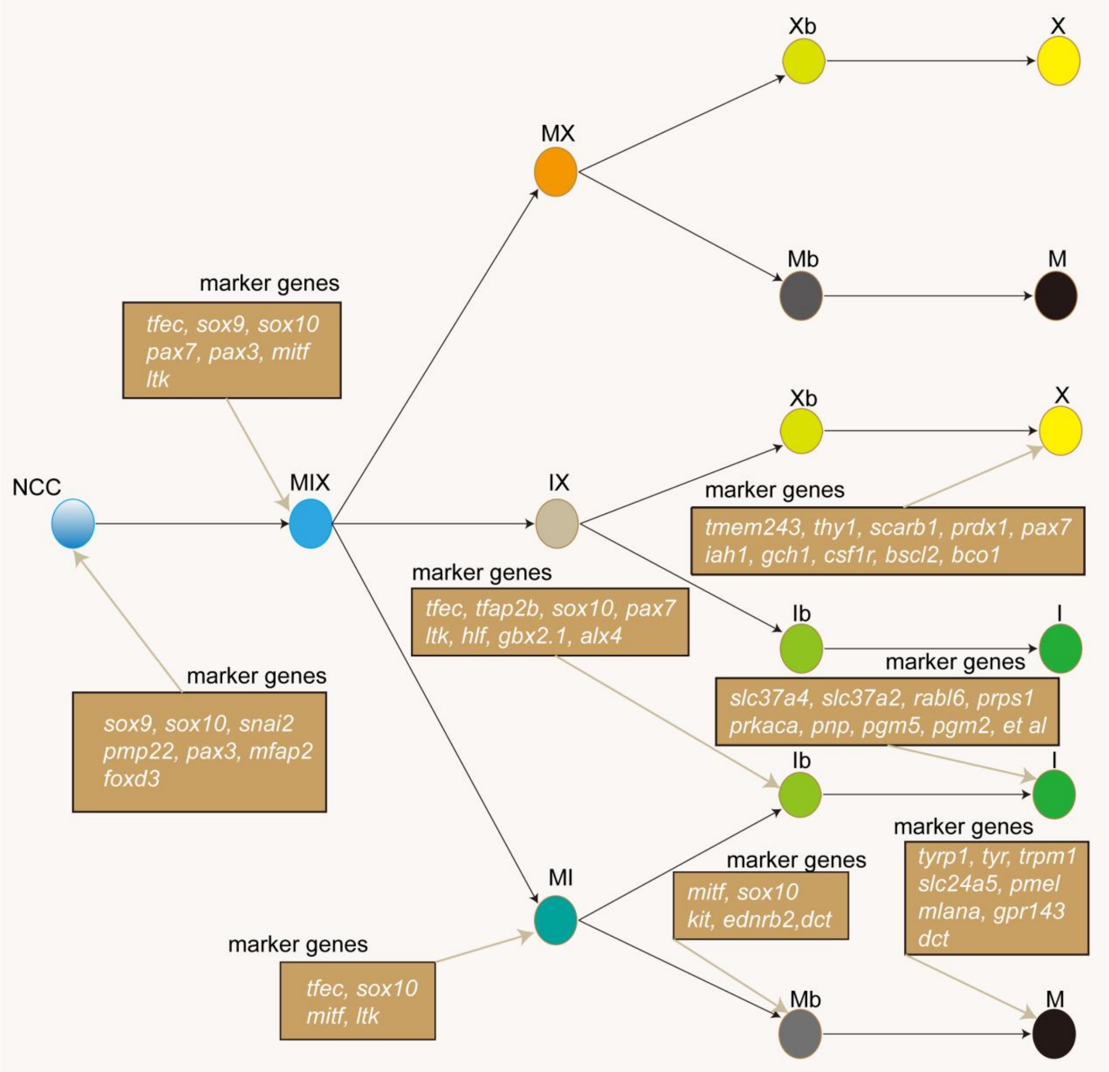

Figure S9. Developmental process of vertebrate xanthophores, iridophores, and melanocytes. The definitions of NCC, MIX, MI, IX, and MX are provided in the manuscript. Xb refers to xanthoblast, Mb to melanoblast, and Ib to iridoblast. M, I, and X represent melanocytes, iridophores, and xanthophores, respectively. The pathways showing the direct differentiation of NCC into Mb, Ib, and Xb, and their subsequent development into melanocytes, iridophores, and xanthophores, are not depicted in the figure.

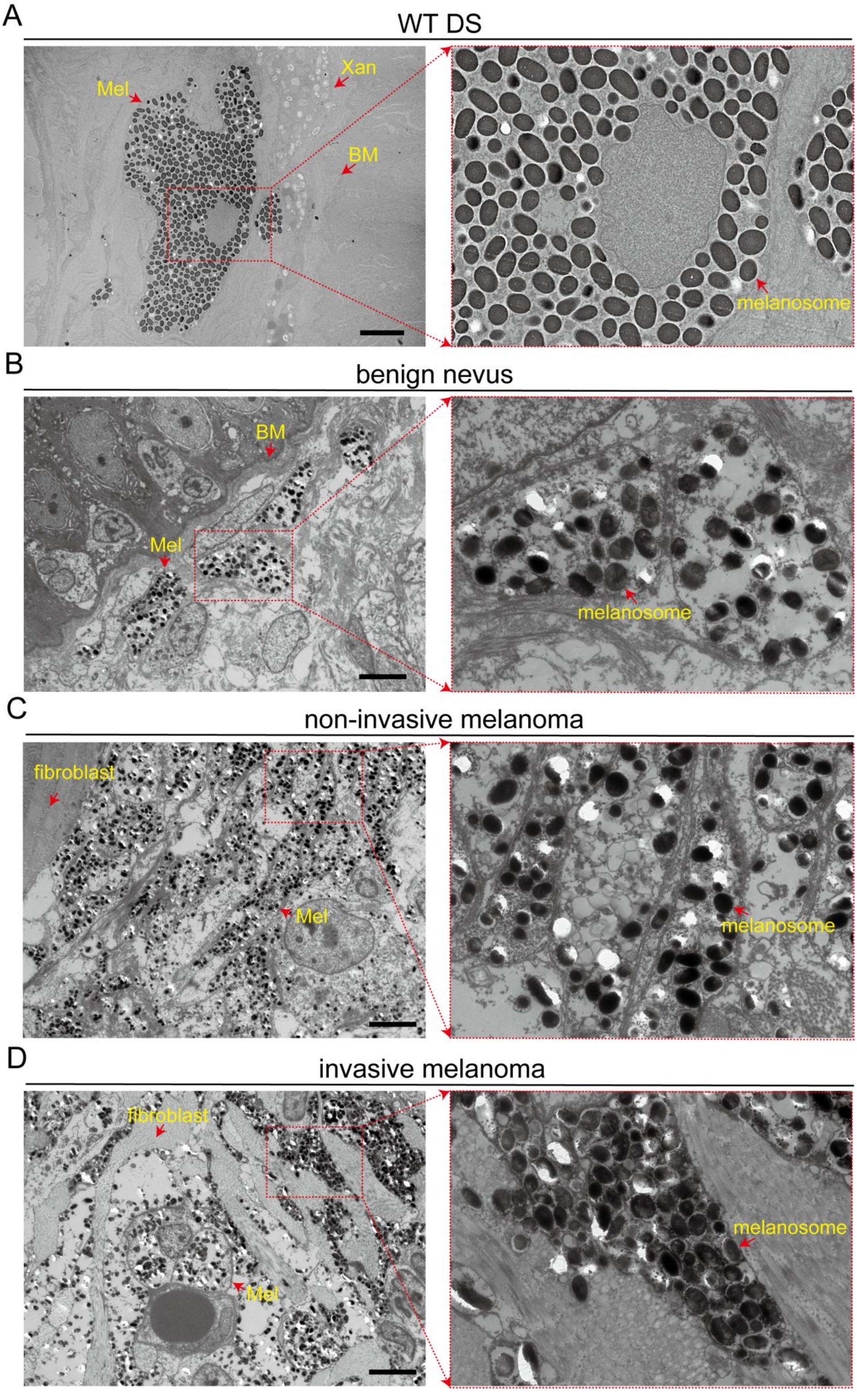
A
WT DS
Mel
Xan
BM
melanosome
B
benign nevus
BM
Mel
melanosome
C
non-invasive melanoma
fibroblast
Mel
melanosome
D
invasive melanoma
fibroblast
Mel
melanosome

Figure S10. Melanophores exhibit significant proliferation during melanoma development in *cdkn2b$^{-/-}$/tp53$^{-/-}$ Xenopus tropicalis*. Panels (A-D) present transmission electron microscopy results of 18-month-old wild-type (WT) *Xenopus tropicalis* dorsal skin (DS), and benign nevus, non-invasive melanoma, invasive melanoma in 18-month-old *cdkn2b$^{-/-}$/tp53$^{-/-}$ Xenopus tropicalis*, respectively. These results suggest a marked increase in melanophore proliferation during melanoma progression. Each group consisted of samples from 3 frogs, with 3 resin-embedded electron microscopy samples prepared per group for ultra-thin sectioning and transmission electron microscopy analysis. The images depict representative transmission electron microscopy results, with 'Mel' representing melanophores, 'Xan' indicating xanthophores, and 'BM' indicating the basement membrane. Scale bars in the images are 5 μm.

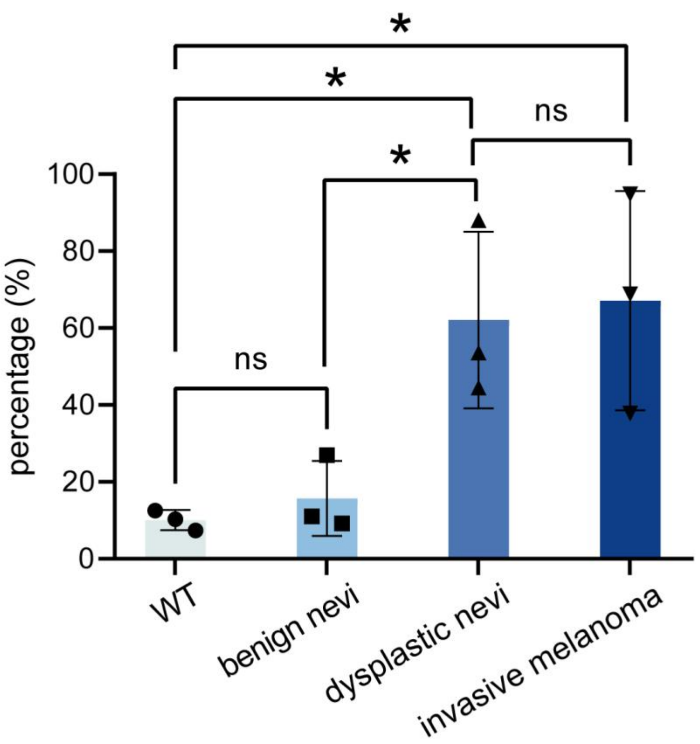

Figure S11. Statistical results of transmission electron microscopy presented in Supplementary Figure 10. Under identical imaging conditions, the percentage of the area occupied by melanocytes relative to the total image area was calculated. Each group comprised data from three distinct photographs. Statistical analyses were conducted using ordinary one-way ANOVA; "ns" indicates no significant difference, * denotes a P-value < 0.05, ** indicates a P-value < 0.01, *** indicates a P-value < 0.001, and **** indicates a P-value < 0.0001.

Supplementary information

tp53 genotyping forward primer	TCAGAAGCCTTTGTTGGAGAGTAG
tp53 genotyping reverse primer	GAATAAAAGAAACGATTACCATCCC
Trac-reverse primer	AAAAGCACCGACTCGGTGCCAC
cdkn2b gRNA forward primer	GAATTCTAATACGACTCACTATAGGGAGTTTGTGGCATTTACAGTTTTAGAGCTAGAAATAGCAAGTT
gRNA scaffold	GTTTTAGAGCTAGAAATAGCAAGTTAAAATAAGGCTAGTCCGTTATCAACTTGAAAAAGTGGCACCGAGTCGGTGCTTTT
cdkn2b genotyping forward primer	CACTGTAACAGAATGGGTAC
cdkn2b genotyping reverse primer	TTTAGCTAGCTAGCATCACC

Table S1. The primers (from 5' to 3' end) used in this project.

Genotype	18 months			
	benign nevi	dysplastic nevi	non-invasive melanomas	invasive melanomas
cdkn2b$^{+/+}$/*tp53*$^{+/+}$	0	0	0	0
cdkn2b$^{+/-}$/*tp53*$^{+/+}$	0	0	0	0
cdkn2b$^{-/-}$/*tp53*$^{+/+}$	2 (100%)	0	0	0
cdkn2b$^{+/+}$/*tp53*$^{+/-}$	3 (60%)	2 (40%)	0	0
cdkn2b$^{+/-}$/*tp53*$^{+/-}$	3 (43%)	3 (43%)	1 (14%)	0
cdkn2b$^{-/-}$/*tp53*$^{+/-}$	3 (50%)	2 (33%)	1 (17%)	0
cdkn2b$^{+/+}$/*tp53*$^{-/-}$	2 (67%)	1 (33%)	0	0
cdkn2b$^{+/-}$/*tp53*$^{-/-}$	1 (20%)	3 (60%)	1 (20%)	0
cdkn2b$^{-/-}$/*tp53*$^{-/-}$	2 (14%)	2 (14%)	2 (14%)	8 (57%)

Table S2. The penetrance of spontaneous benign nevi, dysplastic nevi,

non-invasive melanomas, and invasive melanomas in *Xenopus tropicalis* at 18 months for each genotype.

genotype	pancreatic cancer	lung cancer	liver cancer	sarcoma	splentic tumor	stomach cancer	ovarian cancer
$cdkn2b^{-/-}/tp53^{+/+}$	0/5	0/5	0/5	0/5	0/5	0/5	0/5
$cdkn2b^{+/+}/tp53^{-/-}$	1/6	0/6	0/6	0/6	0/6	0/6	0/6
$cdkn2b^{-/-}/tp53^{+/-}$	0/5	0/5	1/5	1/5	0/5	0/5	0/5
$cdkn2b^{+/-}/tp53^{-/-}$	4/10	2/10	1/10	3/10	1/10	1/10	0/10
$cdkn2b^{-/-}/tp53^{-/-}$	0/6	0/6	0/6	2/6	0/6	0/6	1/6

Table S3. Spontaneous tumors in 40-month-old *Xenopus tropicalis* under different knockout backgrounds of *cdkn2b* and *tp53*, excluding melanoma. The data in the table are preliminary statistics of tumors other than melanomas obtained from dissecting various genotypes of frogs with visibly poor health. $cdkn2b^{-/-}/tp53^{-/-}$ *Xenopus tropicalis* began experiencing mortality around 18 months, and the table reflects examination results from only the surviving 6 frogs.

Table S4. (Provided in an individual Excel file) BeNeVS-Ctr refers to adjacent tissues of benign nevi in the abdomen, BeNeVS denotes benign nevi in the abdomen, DyNeVS stands for dysplastic nevi in the dorsum, MeMeDS represents invasive melanoma in the dorsum, and MeMeDS-Ctr indicates adjacent tissues of invasive melanoma.
